# Mechanistic insight into the RNA-stimulated ATPase activity of tick-borne encephalitis virus helicase

**DOI:** 10.1016/j.jbc.2022.102383

**Published:** 2022-08-17

**Authors:** Paulina Duhita Anindita, Marco Halbeisen, David Řeha, Roman Tuma, Zdenek Franta

**Affiliations:** Department of Chemistry, Faculty of Science, University of South Bohemia, České Budějovice, Czech Republic

**Keywords:** tick-borne encephalitis virus, flavivirus, viral protein, nonstructural protein 3, crystal structure, RNA helicase, ATPase, enzyme kinetics, molecular dynamics, DENV, Dengue virus, HCV, hepatitis C virus, MD, molecular dynamics, NS3, nonstructural protein 3, PDB, Protein Data Bank, Pi, inorganic phosphate, SF2, superfamily 2, TBEV, tick-borne encephalitis virus, TLS, translation/libration/screw, ZIKV, Zika virus

## Abstract

The helicase domain of nonstructural protein 3 (NS3H) unwinds the double-stranded RNA replication intermediate in an ATP-dependent manner during the flavivirus life cycle. While the ATP hydrolysis mechanism of Dengue and Zika viruses NS3H has been extensively studied, little is known in the case of the tick-borne encephalitis virus NS3H. We demonstrate that ssRNA binds with nanomolar affinity to NS3H and strongly stimulates the ATP hydrolysis cycle, whereas ssDNA binds only weakly and inhibits ATPase activity in a noncompetitive manner. Thus, NS3H is an RNA-specific helicase, whereas DNA might act as an allosteric inhibitor. Using modeling, we explored plausible allosteric mechanisms by which ssDNA inhibits the ATPase *via* nonspecific binding in the vicinity of the active site and ATP repositioning. We captured several structural snapshots of key ATP hydrolysis stages using X-ray crystallography. One intermediate, in which the inorganic phosphate and ADP remained trapped inside the ATPase site after hydrolysis, suggests that inorganic phosphate release is the rate-limiting step. Using structure-guided modeling and molecular dynamics simulation, we identified putative RNA-binding residues and observed that the opening and closing of the ATP-binding site modulates RNA affinity. Site-directed mutagenesis of the conserved RNA-binding residues revealed that the allosteric activation of ATPase activity is primarily communicated *via* an arginine residue in domain 1. In summary, we characterized conformational changes associated with modulating RNA affinity and mapped allosteric communication between RNA-binding groove and ATPase site of tick-borne encephalitis virus helicase.

Tick-borne encephalitis virus (TBEV) is an enveloped single-stranded positive-sense RNA virus from the family *Flaviviridae*, genus *Flavivirus*. The virus is neurotropic and causes tick-borne encephalitis, which affects primarily adult population within European and North-Eastern Asian countries (1). While TBEV vaccines are available, because of the lack of targeted campaigns, the actual vaccination coverage is low even in high-risk areas. There is currently no specific treatment available (2, 3). Therefore, to develop antivirals against TBEV, it is essential to understand the structure and function of the key viral enzymes that are involved in the replication.

TBEV encodes three structural and seven nonstructural (NS) proteins. Among the NS proteins, NS3 is a multifunctional protein that comprises two functional domains: a protease and a helicase. The chymotrypsin-like serine protease is located with the N-terminal region (172 amino acids) and is responsible for viral polyprotein processing. This domain is connected by a short flexible linker to the C-terminal helicase domain (NS3H, 434 amino acids), which exhibits several activities: NTPase, RNA helicase, and RNA 5′-triphosphatase. The RNA helicase activity uses energy from NTP hydrolysis for unwinding of dsRNA replication intermediates, whereas RNA 5′-triphosphatase removes the terminal γ-phosphate from the 5′-triphosphate end of the positive-sense ssRNA before mRNA capping by the methyl transferase domain of NS5 protein (4, 5).

In this study, we focus on NS3H, which is a monomeric enzyme belonging to the DEAD/H box subfamily within the helicase superfamily 2 (SF2) (6, 7) and consists of three subdomains. Subdomains 1 and 2 both exhibit the highly conserved RecA-like fold typical of P-loop NTPases. The NTPase active site is located in a cleft between the subdomains 1 and 2 and involves motifs I (Walker A), II (Walker B), and VI. A groove between subdomain 3 and subdomain 1 and 2, respectively, forms the RNA-binding site. The coupling between NTPase-binding and RNA-binding sites is essential for the helicase activity (8, 9).

Several structural studies of flavivirus helicases from Dengue virus (DENV), Zika virus (ZIKV), yellow fever virus, Kunjin virus, and Japanese encephalitis virus revealed high structural conservation among them (10, 11, 12, 13, 14). In addition, previous biochemical and structural studies have provided insight into substrate binding and revealed structural changes associated with the ATP hydrolysis cycle and identified RNA-interacting residues for the DENV and ZIKV helicases (11, 15, 16, 17). Recent molecular dynamics (MD) simulations (9, 18) provided further insight into conformational changes associated with RNA binding and stimulation of the ATP hydrolysis, which was considered the rate-limiting step. In contrast, inorganic phosphate (Pi) release has been established as the rate-limiting step for hepatitis C virus (HCV) NS3 helicase that is considered a mechanistic model system for viral SF2 (19). Hence, it remains to be seen whether Pi release or the hydrolysis is the rate-limiting step and whether the coupling and the associated allosteric changes are conserved among all SF2 helicases and between helicases from different flaviviruses.

Here, we biochemically characterized NS3H from TBEV and obtained structures of key intermediates along the ATPase cycle (nucleotide-free, apo; ADP-; adenylyl-imidodiphosphate, AMPPNP-; and hydrolysis product, ADP–Pi-bound NS3H). While the overall structure of the TBEV NS3H is closely related to that of DENV and ZIKV, structural variation is observed in different nucleotide states. Trapping of ATP hydrolysis products within the crystal and the negligible basal activity (*i.e.*, in the absence of RNA) suggest that the ATPase rate-limiting step is phosphate release, which is allosterically stimulated by RNA. Indeed, ssRNA binds to NS3H with nanomolar affinity and stimulates its ATPase activity, whereas ssDNA inhibits the ATPase activity without directly competing with RNA. We explored ssRNA binding by modeling and MD simulations and identified conserved RNA-binding residues. We examined the allosteric roles of these residues by site-directed mutagenesis and functional assays. These results and simulations suggest that RNA binding is relayed to the ATPase active site *via* a conserved residue within domain 1, whereas RNA affinity is modulated by conformational changes associated with opening and closing of the ATP-binding cleft.

## Results and discussion

### RNA-stimulated NTPase activity of recombinant NS3H is inhibited by DNA

**Figure 1:**
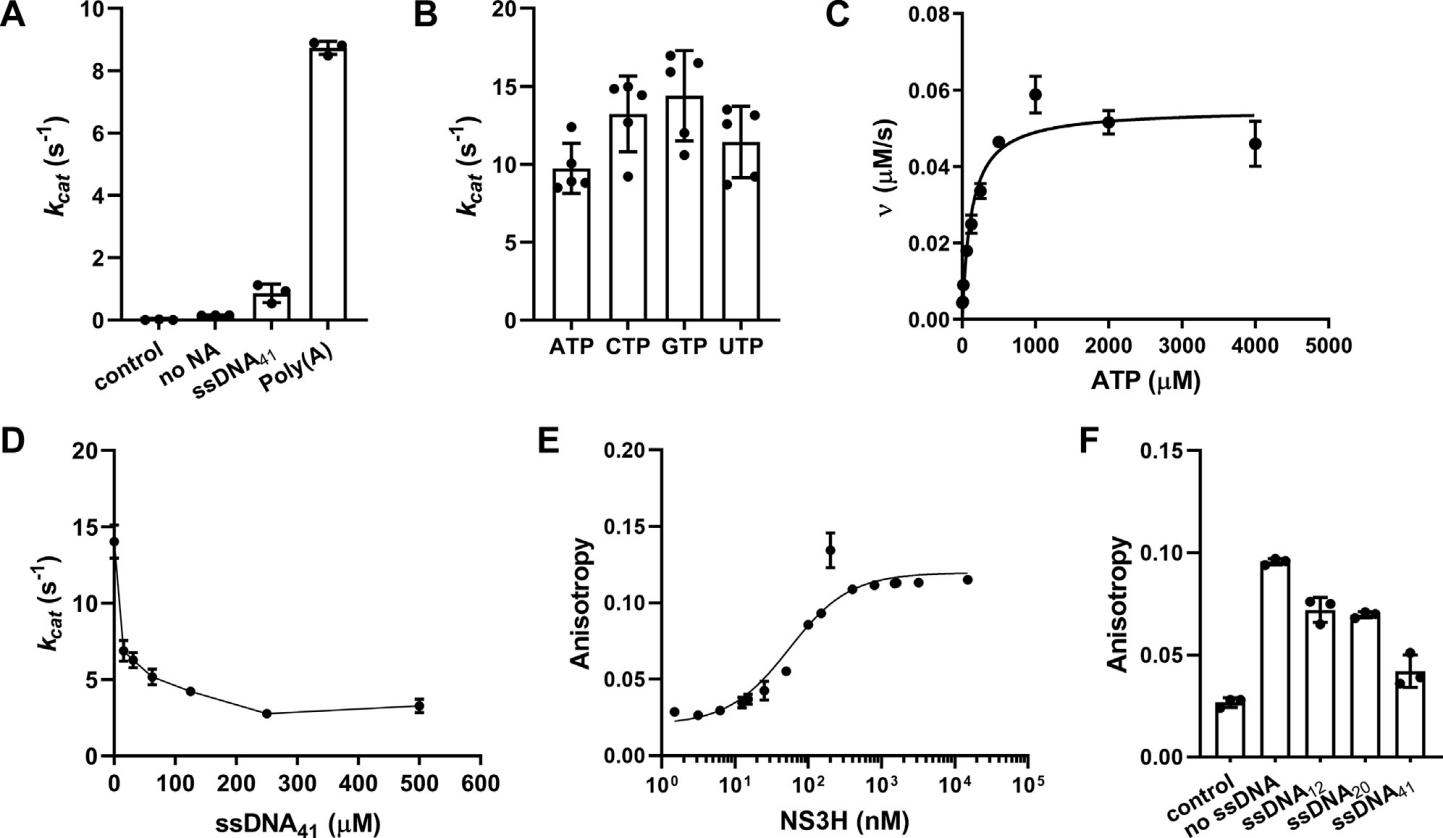
**ATPase activity of recombinant NS3H.***A*, ATPase activity of 6.25 nM NS3H was stimulated by 1.4 mM (nucleotide concentration) poly(A) in the presence of 1 mM ATP. The presence of 1 mM ssDNA_41_ did not show similar stimulation. The possible free Pi contamination from ATP in the absence of NS3H (control) and ATP hydrolysis by NS3H in the absence of any nucleic acid (no NA) were measured. *B*, NS3H hydrolyzed 1 mM NTP substrates (ATP, CTP, GTP, and UTP, respectively) in the presence of poly(A). *C*, Michaelis–Menten plot of phosphate release velocity in the presence of 6.25 nM NS3H for ATP substrate (0–4000 μM). *D*, concentration-dependent inhibition of RNA-stimulated ATPase activity by ssDNA_41_ (15.625–500 μM). *E*, binding of NS3H to 6-FAM-ssRNA_12_ measured by fluorescence anisotropy. The measurement was done using 10 nM 6-FAM-ssRNA_12_ and increasing the concentration of NS3H from 1.5 nM to 15 μM. *F*, inhibition of 6-FAM-ssRNA_12_ binding to NS3H by 800 nM ssDNA_12_, ssDNA_20_, or ssDNA_41_ was monitored using fluorescence anisotropy. The anisotropies of 6-FAM-ssRNA12 in the absence (control) and presence of NS3H (no ssDNA) were measured as controls. Data are plotted as mean ± SD. NS3, nonstructural protein 3; Pi, inorganic phosphate.

The recombinant NS3H protein fused with N-terminal 10X-histidine tag was purified to homogeneity (Fig. S1). To assess the ATPase activity of NS3H, a phosphate release assay was performed in the absence and presence of nucleic acids, ssRNA or ssDNA. ATPase activity of NS3H was strongly dependent on the presence of RNA substrate, poly(A), whereas the basal ATPase activity was negligible at the same protein concentration (Fig. 1*A*). NS3H hydrolyzed all four NTP substrates with comparable turnover (Fig. 1*B*). This demonstrated that NS3H exhibits little specificity for different NTP substrates, as shown previously for DENV helicase (20). Basic steady-state kinetic parameters for ATP were determined in the presence of poly(A) (Fig. 1*C*). The Michaelis–Menten constant *K*_*m*_ = 125 ± 15 μM and turnover *k*_cat_ = 8.8 ± 0.2 s^−1^ of NS3H are similar to those of DENV helicase in the presence of short 5′-UTR RNA fragment (15).

While ssDNA fails to stimulate ATPase activity significantly (Fig. 1*A*), it can partially inhibit it in a dose-dependent manner at micromolar concentrations (Fig. 1*D*). However, these micromolar concentrations are much higher than the affinity for ssRNA (apparent *K*_*D*_ = 49.7 ± 9.3 nM), which was determined by fluorescence anisotropy–based binding assay (Fig. 1*E*). Using the same approach, we observed that ssDNA partially competes with RNA binding at ∼20-fold molar excess of apparent *K*_*D*_ for ssRNA substrate. Longer ssDNAs inhibited RNA binding more effectively (Fig. 1*F*), suggesting that the longer DNA might bind to several weak nonspecific binding sites. Altogether, these results indicate that the binding of the helicase to RNA substrate stimulates ATP hydrolysis, and DNA may act as a noncompetitive inhibitor *via* nonspecific binding. This specificity of NS3H toward RNA substrate is different from HCV helicase (21) and DENV helicase (22), which, in addition to RNA binding, also exhibit DNA binding with affinities ranging from nanomolar to micromolar range.

### TBEV NS3H structure is similar to that of other flavivirus helicases

**Figure 2:**
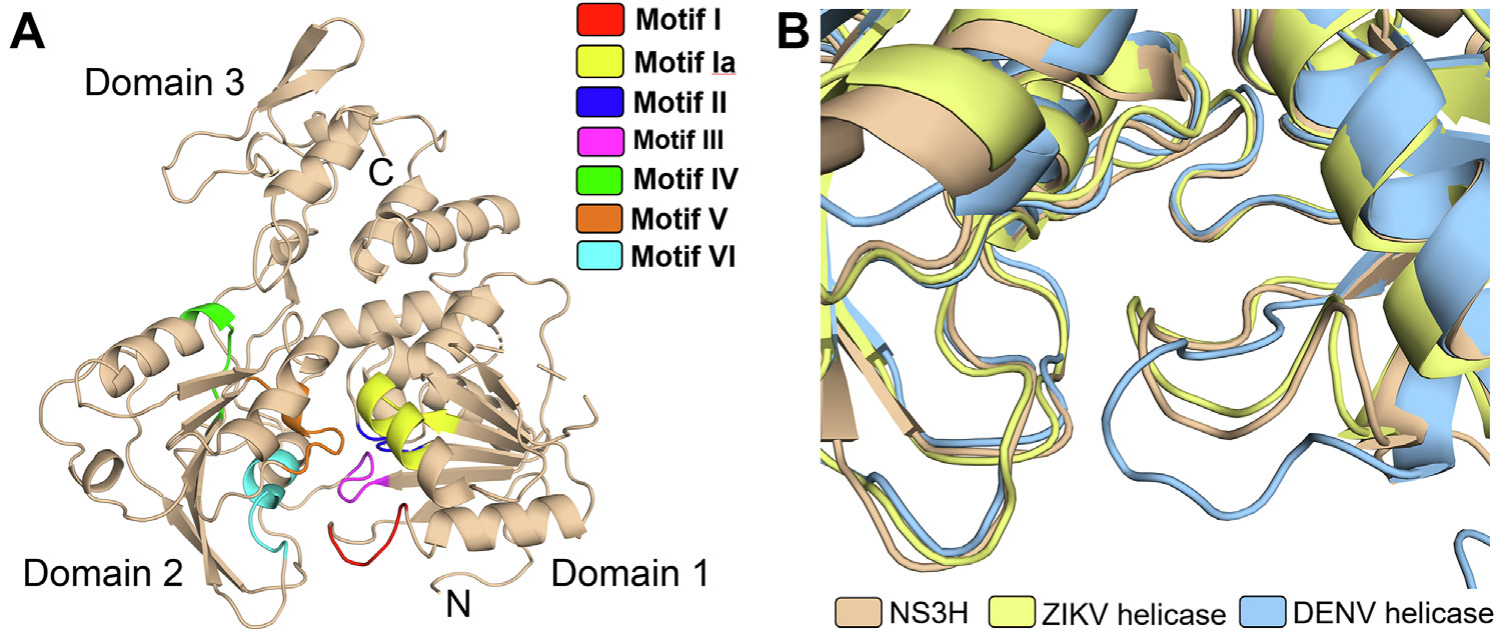
**Overall structure of apoNS3H and structural comparison with DENV and ZIKV helicases.***A*, a 3-dimensional structure of apoNS3H is shown in *cartoon* representation. SF2 helicase motifs are highlighted in *colors*. The N and C termini are labeled. *B*, superposition of NS3H (*wheat*) with DENV (*light blue*) and ZIKV (*pale yellow*) helicases showing ATPase site. NS3H adapts similar P-loop conformation as ZIKV helicase. DENV, Dengue virus; NS3, nonstructural protein 3; SF2, superfamily 2; ZIKV, Zika virus.

The apoNS3H protein crystallizes in space group *P*4_1_2_1_2 at 1.83 Å (Table 1). The refined model of apoNS3H (residues 173–621) is complete except for an N-terminal 10X-histidine tag region derived from the pET-19b vector together with a short flexible linker region between protease and helicase domains of NS3 (residues E173–Q181) and two nonconserved disordered loop regions (residues P251–G259 and T502–P506). Nonprotein positive *2F*_o_*–F*_*c*_ electron density was attributed to water molecules. The resulting 3D protein structure shows overall similarity with already published helicase structures of other flaviviruses (10, 11, 12, 13, 14, 16, 17, 23). NS3H has a clover-shaped architecture divided into three domains containing seven conserved motifs of SF2 helicase family (Figs. 2*A* and S2). The Rec-A-like domains 1 and 2 adopt the α/β open sheet topology (Rossman fold) (5). Domain 1 (residues W188–E329) consists of six β-strands surrounded by four α-helices, whereas domain 2 (residues P330–G486) consists of three α-helices and six β-strands with one antiparallel β hairpin passing close to domain 3. Domain 3 (residues L487–R621) consists of four α-helices and one β-hairpin. To our knowledge, the apoNS3H crystal structure derived from the TBEV MucAr-HB-171/11 virus strain has also been reported elsewhere (7); however, the coordinates have not been deposited in Protein Data Bank (PDB). A similar crystal structure of apoNS3H from TBEV strain HYPR is also available in the PDB (code: 7JNO) with the overall RMSD of 0.8 Å against our apoNS3H calculated using DALI server (GNU General Public License) (24).

**Table 1:** Crystallographic data collection and refinement statistics

Dataset	Apo	ADP–Mn^2+^	AMPPNP–Mn^2+^	ADP–Pi–Mn^2+^
PDB code	7OJ4	7BLV	7BM0	7NXU
Data collection				
Space group	*P* 4_1_ 2_1_ 2	*P* 4_1_ 2_1_ 2	*P* 4_1_ 2_1_ 2	*P* 4_1_ 2_1_ 2
Unit cell parameters				
*a = b, c* (Å), α = β = γ (°)	73.30, 196.05, 90.00	73.12, 196.13, 90.00	72.95, 196.45, 90.00	72.98, 196.59, 90.00
Resolution range (Å)^a^	49.06–1.83 (1.94–1.83)	45.78–2.10 (2.23–2.10)	45.71–1.90 (2.00–1.90)	48.81–2.10 (2.22–2.10)
Unique reflections^a^	48,205 (7581)	32,056 (5054)	43,006 (6796)	31,603 (5033)
Multiplicity^a^	24.54 (17.03)	14.31 (13.77)	14.25 (14.64)	13.57 (13.42)
Completeness (%)^a^	99.9 (99.3)	99.9 (99.7)	99.9 (99.7)	98.4 (99.5)
I/δ^a^	28.87 (2.22)	24.38 (2.53)	19.10 (2.03)	23.02 (3.21)
*R*_meas_ (%)^a^	7.5 (126.6)	6.7 (113.3)	8.6 (118.1)	9.9 (83.7)
Wilson *B*-factor (Å^2^)	34.3	47.1	35.6	34.2
Refinement				
*R*_work_/*R*_free_^b^ (%)	18.1/22.9	23.3/25.9	21.6/22.9	21.4/25.1
Average *B*-factor (Å^2^)				
Overall	46	57	42	42
Protein	46	57	42	41.3
Ligands	—	100.8 (ADP)	84 (AMPPNP)	90.7 (ADP), 31.8 (PO_4_)
No. of non-H atoms				
Protein	3375	3414	3389	3357
Water	209	75	230	200
Metal ions	—	1 (Mn^2+^)	1 (Mn^2+^), 1 (Na^+^)	1 (Mn^2+^), 1 (Na^+^)
Ligands	—	27 (ADP)	31 (AMPPNP)	27 (ADP), 5 (PO_4_)
RMSDs				
Bond length (Å)	0.0146	0.0107	0.0155	0.00091
Bond angles (°)	1.929	1.619	1.765	1.540
Ramachandran plot				
Favored (%)	97	95	97.37	97
Allowed (%)	3	5	2.63	12
Outliers (%)	0	0	0	0

a Values in parentheses are for the highest resolution shell.

b *R*_free_ was calculated using 5% of the data excluded from refinement.

The NTPase site is situated at the interface between domains 1 and 2. It encompasses Walker A and Walker B motifs identified in domain 1 and motif VI in domain 2. Walker A motif consists of G_202_SGKT_206_ sequence forming the P-loop region of NTPase site with residue K205 known to recognize the β- or γ-phosphate of ATP in homologous helicases (12). Walker B motif contains a conserved D_290_EAH_293_ sequence, which is common among flavivirus helicases (reviewed in Ref. (4)). Motif VI (Q_459_RRGRVGR_466_) includes the arginine fingers (R463 and R466), which are important in the energy coupling during the NTP hydrolysis (12, 25) and in recognizing the γ-phosphate of ATP (13). The empty NTPase site is filled with solvent molecules, and the P-loop adopts a “relaxed” conformation as found in ZIKV structure (10), whereas DENV helicase exhibits yet more open conformation (11) (Fig. 2*B*).

### Structural snapshots of ATP hydrolysis cycle

Three ternary complexes of NS3H with AMPPNP–Mn^2+^, ADP–Pi–Mn^2+^, or ADP–Mn^2+^, respectively, were captured in crystallographic structures. Based on these structures, NS3H seems to follow a common mechanism of ATP hydrolysis cycle in flaviviruses (11, 16). Here, the hydrolysis cycle is represented by four states: (i) a prehydrolysis, AMPPNP–Mn^2+^-bound state representing the binding of ATP molecule to the helicase; (ii) a post-hydrolysis intermediate, ADP–Pi–Mn^2+^-bound state; (iii) a product dissociation intermediate with Pi released and ADP–Mn^2+^ are still bound to the protein; and (iv) nucleotide-free (apo) product release state, which is ready to bind ATP.

**Figure 3:**
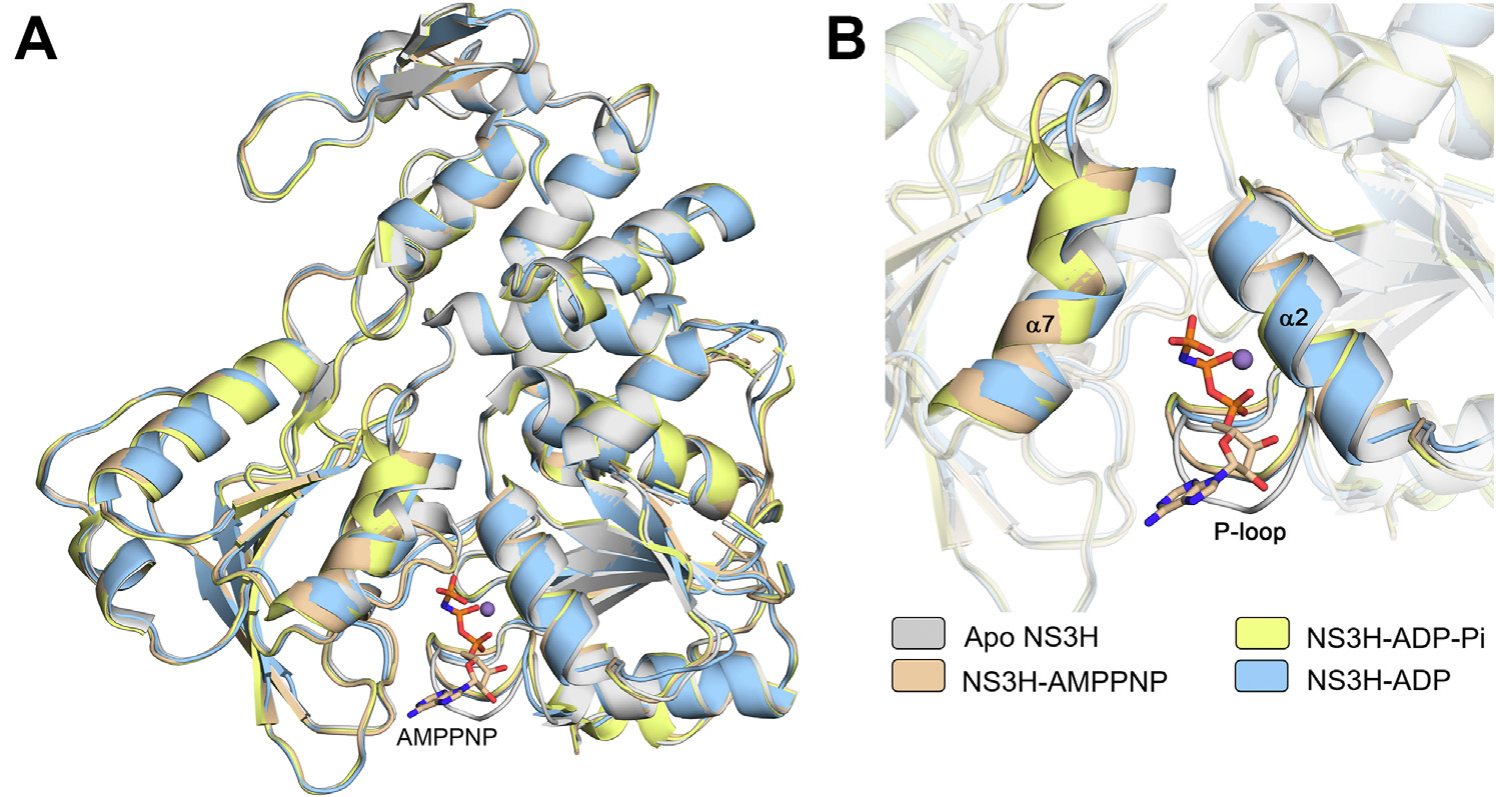
**Nucleotide-bound complexes.***A*, superposition of apo and ternary NS3H structures showing global protein conformation. *B*, close-up view of ATPase site corresponding to (*A*) where α2, α7, and P-loop are highlighted. A conformational change in the tip of α7 and P-loop is observed with respect to the bound-ligand. AMPPNP (*stick representation*) and manganese ion (*purple sphere*) are shown. NS3, nonstructural protein 3.

**Figure 4:**
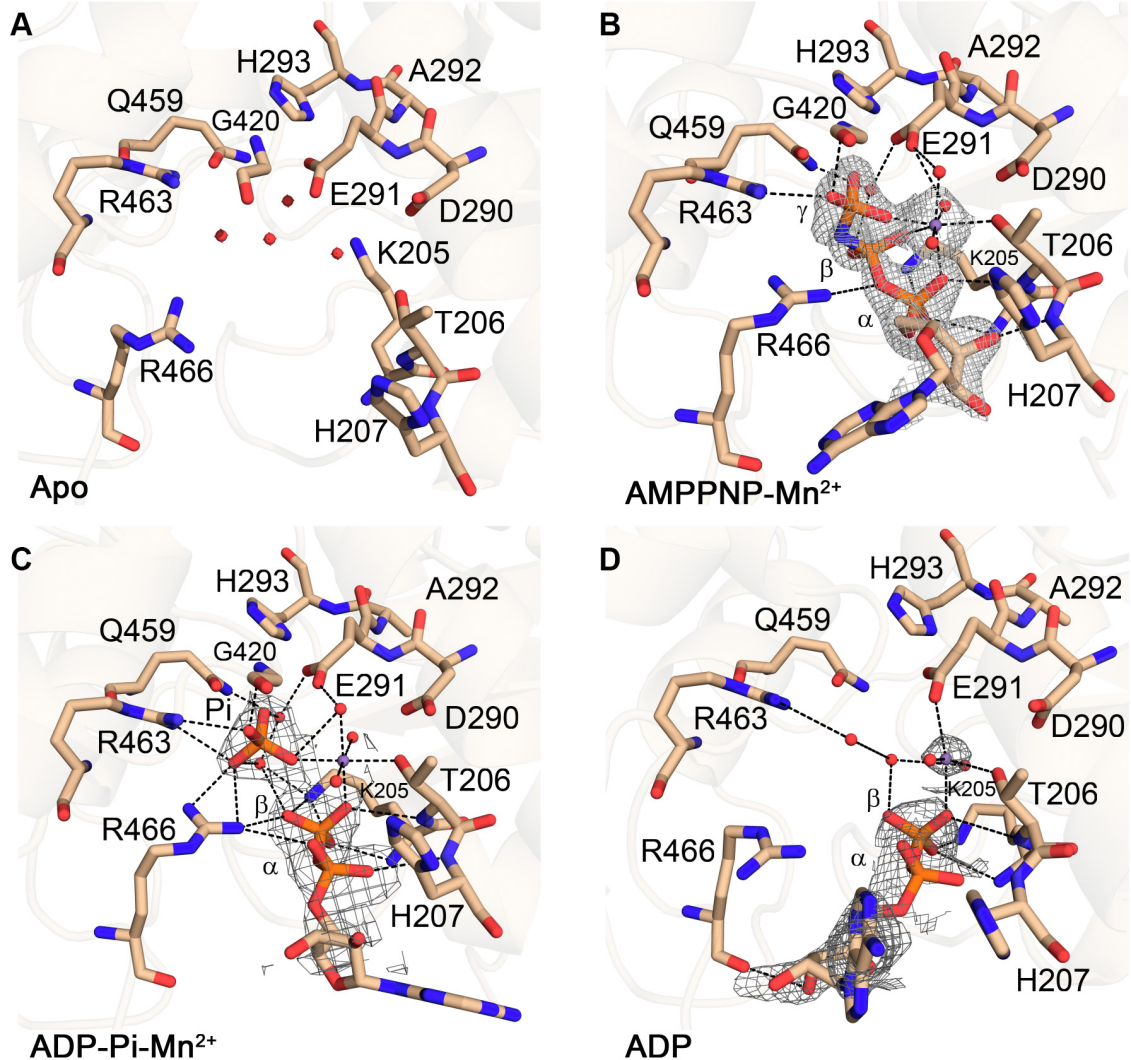
**Interactions within nucleotide-binding site in apo and nucleotide-bound complexes.** Important residues in the NTPase site are shown in the *stick* representation. (*A*) apoNS3H, (*B*) AMPPNP–Mn^2+^ ternary complex, (*C*) the ADP–Pi–Mn^2+^ ternary complex, and (*D*) the ADP–Mn^2+^ ternary complex. The manganese ion and water molecules are represented as *purple spheres* and *red spheres*, respectively. Hydrogen bonds and metal ion coordination are displayed as *black dashed lines*. The difference Fourier map shows nucleotide ligands at level of 1σ and manganese ions at level of 2σ (all in *gray mesh*). NS3, nonstructural protein 3.

The AMPPNP–Mn^2+^- and ADP–Mn^2+^-bound NS3H structures were obtained *via* cocrystallization of apoNS3H with corresponding nucleotides, whereas the ADP–Pi–Mn^2+^ complex was obtained as a result of ATP hydrolysis during crystallization, that is, Pi remained trapped within the structure. The nucleotide complexes crystallize in *P*4_1_2_1_2 space group with one molecule in the asymmetric unit similar to the apoNS3H (Table 1). Hence, structural changes seen in the ternary complexes are not likely because of differences in crystal contacts. Several localized conformational changes within individual domains were observed (Fig. 3*A*) when the nucleotide complexes were compared with apoNS3H. Upon nucleotide and divalent ion (Mn^2+^) binding, the P-loop swings toward the bound nucleotide (Fig. 3*B*) with an inward orientation of K_205_ side chain to coordinate the phosphate group (Fig. 4). In AMPPNP–Mn^2+^ and ADP–Pi–Mn^2+^ complexes, the tip of α7 helix moves away from the relative position in apoNS3H (Fig. 3*B*) to accommodate γ-phosphate. This motion is absent in the ADP–Mn^2+^ structure.

In the prehydrolysis state, NS3H–AMPPNP–Mn^2+^ complex (Fig. 4*B*), the triphosphate moiety of AMPPNP interacts with P-loop residues K205 and T206 *via* hydrogen bonds and with residues D290 and E291 of Walker B motif and Q459 of motif VI *via* water-mediated coordination. One of the arginine fingers, R463, and residue G420 (motif V), also interacts with γ-phosphate of AMPPNP. The 3′-OH group of the ATP ribose C3′ endo ring pucker is hydrogen-bonded with the main chain amide nitrogen in between residues T206 and H207. One potentially catalytic water molecule coordinates with the side chains of E291 (Walker B), Q459 (motif VI), and γ-phosphorus atom in location suggested by AMPPNP-bound DENV helicase (11) and ATP-bound ZIKV helicase structures (16). The triphosphate moiety of AMPPNP is in a staggered conformation (as defined in Ref. (26)), whereas in DENV helicase (PDB code: 2JLR) (11), it adopts a coplanar/eclipsed conformation (Fig. S3), which is proposed to facilitate nucleophilic attack during hydrolysis (27).

The NS3H–ADP–Pi–Mn^2+^ complex (Fig. 4*C*) was captured following the cocrystallization of NS3H and ATP, implying that ATP was hydrolyzed during crystallization or within the crystal and that the subsequent Pi release is extremely slow given the lengthy crystallization protocol. This suggests that phosphate release is the rate-limiting step of the ATPase cycle (in the absence of RNA) and is prevented by the closed conformation of the ATP-binding site. The low basal ATPase activity, as measured by phosphate release assay in the absence of RNA, further indicates that this step is facilitated by RNA binding and might be coupled to RNA translocation. This is similar to the proposed HCV mechanism (19). However, recent theoretical results for DENV helicase suggested that ATP hydrolysis is the rate-limiting step, which is accelerated by RNA (9). We observed that both arginine fingers (R463 and R466) and residue G420 (motif V) directly interact with the free Pi, whereas residues Q459 and E291 interact with the free Pi *via* water-mediated coordination. It is worth noting that free Pi occupies the same position as γ-phosphate in the AMPPNP–Mn^2+^ complex, whereas the β-phosphate from the ADP molecule occupies the position of α-phosphate in the AMPPNP–Mn^2+^ complex, which is also in similar position as the one captured in the ADP–Mn^2+^ post-hydrolysis complex (Fig. 4*D*). Thus, the strong interactions of the γ-phosphate are driving nucleoside triphosphate deeper into the binding pocket prior to hydrolysis. Hydrolysis then leads to irreversible phosphate separation, producing a long-lived post-hydrolysis state before Pi and ADP release from the NTPase site.

In the NS3H–ADP–Mn^2+^ complex (Fig. 4*D*), the arginine fingers do not interact directly with any of the phosphate moiety of ADP. Residue R463 coordinates a water molecule, whereas the side chain of residue R466 moves away from the hydrolysis site. In addition, the 3′-OH group of the ribose C3′ endo ring pucker interacts with the main chain carbonyl oxygen of R466. Therefore, this structure captured a state with diminished diphosphate interactions that may facilitate ADP release.

### Conformational changes upon RNA binding

**Figure 5:**
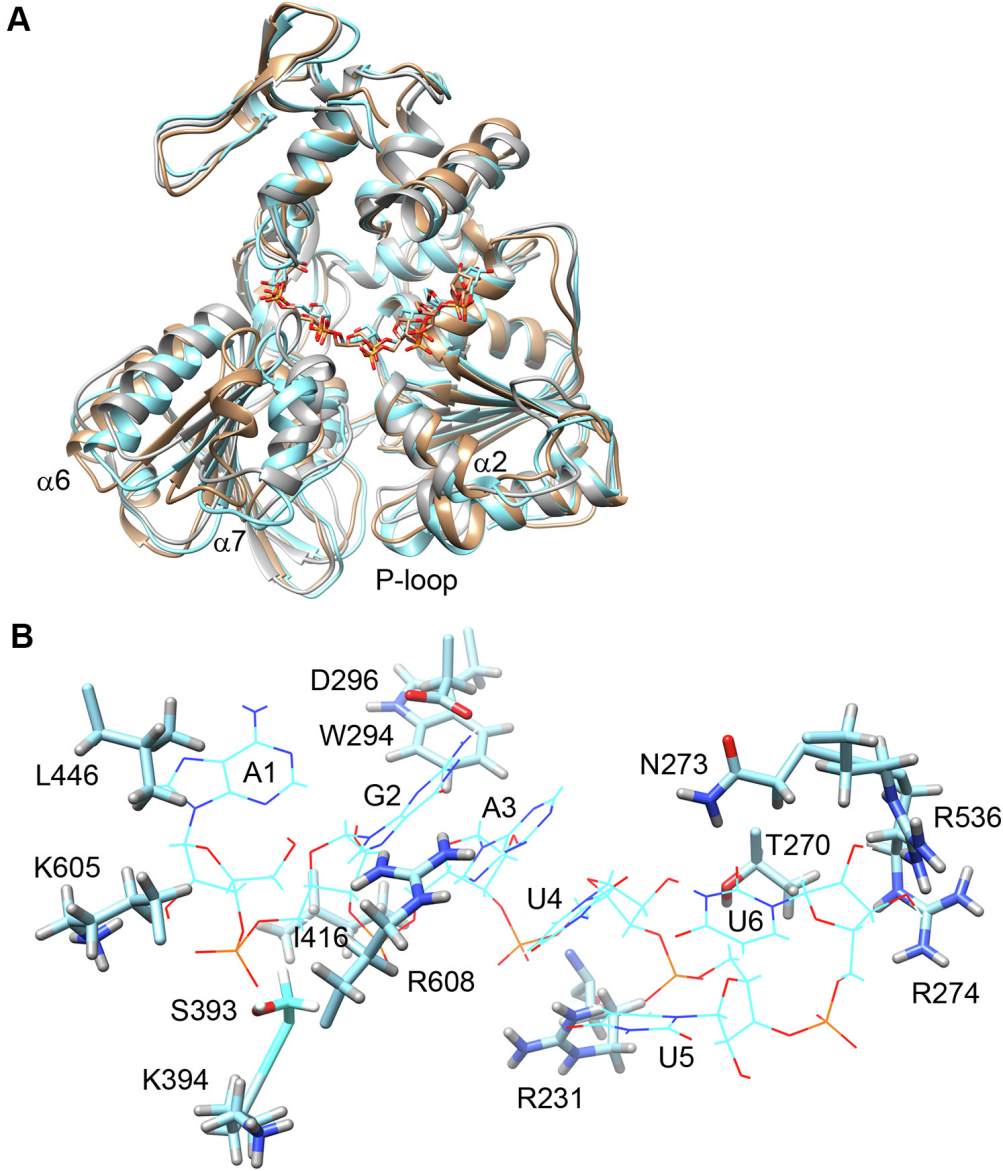
**Modeling RNA binding by MD simulations.***A*, comparison of apoNS3H crystal structure (*gray ribbons*) and two RNA-bound conformations with low (*wheat*) and high (*cyan*) affinity. Bound RNAs are shown as backbone only (two closely related configurations of hexanucleotide). Protein elements that undergo conformational changes associated with RNA binding are labeled in *black*. *B*, configuration of RNA (wireframe) in the binding cleft of the high-affinity conformation. Interacting protein side chains are shown in *stick* representation. Carbon atoms—*cyan*, hydrogen—*light gray*, oxygen—*red*, nitrogen—*blue*, and phosphorus—*orange*. MD, molecular dynamics; NS3, nonstructural protein 3.

Our data and previously published study (22) show that RNA binds with nanomolar affinity and stimulates NS3H ATPase activity. We followed crystallization strategies similar to those previously used for DENV (11) and ZIKV (17) but with no success. To gain further insight into RNA binding, we constructed a model based on the closely related DENV helicase crystal structure (PDB code: 2JLU (11)) with bound (resolved) hexanucleotide ssRNA. RNA oligo was placed into the binding groove by superposing the conserved protein structures. Any clashes in the resulting complex were relieved by molecular mechanics and then subjected to MD simulations (trajectories up to 1 μs) to generate an ensemble of RNA-bound structures (Fig. 5). RNA interaction energies (*E*_int_) were computed for frames along the trajectory using generalized Born surface area method. Frames with diminished *E*_int_ may represent states in which weak interactions between the protein and RNA facilitate relative movement and enable translocation. In addition, structural comparison between states exhibiting high and low *E*_int_, respectively, may reveal how protein conformational changes modulate RNA affinity.

Both protein and RNA quickly depart from the initial model as demonstrated by RMSD (Fig. S4*A*, *left panel*) and sample multiple conformations. Likewise, RNA is bound with widely different *E*_int_ suggesting a substantially dynamic association (Fig. S4*A*, *right panel*). Overall structural differences are illustrated by comparing selected frames with low (*E*_int_ = −150 kJ/mol) and high affinity (*E*_int_ = −350 kJ/mol) conformations, respectively, with the starting apoNS3H structure (Fig. 5*A*). The overall disposition of protein secondary structure elements and motifs is similar between high RNA affinity and apoNS3H structures, whereas the low-affinity conformation differs substantially. With the exception of the P-loop, which assumes conformation seen in nucleotide-containing structures in the presence of RNA, larger conformational changes are mostly confined to domain 2. However, the α2 helix in the low-affinity structure moves closer to the β-sheet core and opens the nucleotide-binding pocket. In domain 2, the α6 helix shifts away from the RNA-binding groove. In the low-affinity structure, this shift is further accentuated by a relative rotation of the whole domain 2. This rotation and translation of α7 helix lead to a widely open nucleotide-binding cleft in the low-affinity structure, whereas in the high-affinity structure, the α7 helix only rotates around its axis. Slight repositioning of domain 3 further opens the RNA-binding cleft in the low-affinity structure.

The overall conformational differences are a result of local interactions between RNA and protein. As shown in Table 2, the interactions are distributed over residues belonging to all three domains. As expected, there are fewer or weaker interactions in the low-affinity structure. In the high-affinity model (Fig. 5*B*), the ssRNA backbone is anchored by interactions between polar or charged residues along the edge of the groove (K605, R231, T270, R536, R274, and S393). The polar group interactions are augmented by van der Waals contact from hydrophobic groups lining the groove (L446, W294, and I416). In addition, there are several base-specific contacts. The G2 base, which is conserved within the 5′-UTR of most flaviviruses (15), is recognized by a hydrogen bond between D296 side-chain carboxy group and the guanine amino group and by two hydrogen bonds from the guanidinium group of R608 to N7 and O2 of the G2 base, respectively. Another specific hydrogen bond is between N273 and O2 of the U6 base, which is also conserved among many but not all flaviviruses.

**Table 2:** RNA interactions by residue and protein conformation

Protein	Atom/group	RNA	Group	Type	High affinity	Low affinity
L446	CD1	A1	Base	VdW (Van der Waals)	+	−
K605	NZH	A1	2′-O	HB	+	−
K394	NZH_3_^+^	G2	P	Salt bridge/ion pair	−	+
R608	NH1/NH2	G2	N7/O6	HB	+	−
S393	OGH	G2	PO	HB	+	+/−
D296	O	G2	NH2	HB (hydrogen bond)	+	+
W294	CH2	A3	H4′	VdW	+	+
I416	CD	A3	2H5′	VdW	+	+/−
T270	OG	U4	2′-OH	HB	+	+
R231	Backbone NH	U4	PO	HB	+	+
R231	NH2	U5	PO	HB/salt bridge	+	+
N273	NDH	U6	O2	HB	+	+/−
R536	NH2	U6	2′-O	HB	+	−
R274	NH2	U6	P	Salt bridge	+	+

Comparison between the pattern of RNA–protein interactions between the low- and high-affinity structures offers insights into possible modulation of RNA affinity by protein conformational changes (Fig. S4*B* and Table 2). In both TBEV models, the bound RNA is in an extended conformation (Fig. S4*B*), similar to that observed in the corresponding DENV crystal structure (Fig. S4*C*). The 5′ end of the RNA backbone is anchored in approximately the same place, but the interacting residues are different. In the high-affinity structure, the G2 5′ phosphate is held in place by K605 (domain 3) and S393 (N-terminal end of α7 helix). The former interaction is replaced by K394 (N-terminal end of α7 helix), and the S393 contact is weakened in the low-affinity structure. The conserved G2 base is shifted in position and only held by D296, and the backbone and base positions downstream are displaced and held by fewer contacts in the low-affinity structure (Fig. S4*B* and Table 2). This suggests that the low-affinity structure may represent an intermediate in which domain 2 moves toward the 5′ end, whereas domain 1 interaction with RNA is weakened and the empty nucleotide-binding cleft is wide open. Closure of the nucleotide-binding cleft upon ATP binding might then cause movement of domain 1 toward the 5′ end, completing an “inchworm” step in the direction of translocation (28).

Based on our models, TBEV NS3H binds RNA in an extended conformation, primarily *via* interactions with the phosphodiester backbone and in a similar fashion as shown for DENV and ZIKV helicases (11, 15, 17). RNA specificity is augmented by interactions of domain 1 T270 with 2′-OH of U4 and domain 3 R536 hydrogen bond to 2′-O of the U6 (Table 2). Furthermore, specific recognition of the 5′-UTR sequence is primarily mediated by three hydrogen bonds (D296, R608) to G2 and augmented by the N273 hydrogen bond to O2 of the U6 base, which is also part of the conserved 5′-UTR motif. Apart from D296, the sequence specific interactions are weakened in the low-affinity conformation, and this is similar to the alternative conformations of R538 seen in crystal structures of DENV helicase with different RNAs (15). Unlike crystal structures, where larger domain motions might be prevented by lattice packing, MD simulations suggest that the protein is quite dynamic in the RNA-bound complex and explores conformations with different RNA affinity and disposition of domains 1 and 2. These configurations may facilitate the handover of RNA between these two domains as proposed by the “inchworm” mechanism (28) and “ratchet mechanism” (29). It remains to be seen whether and how these configurations are related to phosphate release, the rate-limiting step of the ATPase.

While ssRNA is a good model for ATP-driven translocation, the natural substrate is double stranded. In order to model a plausible configuration of dsRNA within the context of the helicase, we have used the MD-equilibrated NS3–ssRNA complex and extended the RNA in both 5′ and 3′ directions and added a partially complementary strand at the 5′ end of the bound oligonucleotide. The double-stranded portion was formed of three 5′ end strong G–C pairs and three weaker A–U base pairs at the junction and a 5′ overhang on the complementary strand to form a fork. After 500 ns, the system was stable, mostly exhibiting RNA fluctuation at the exit (Movie S1). Even after 1 μs simulation, the A–U junction remained intact, reflecting the slow base pair opening dynamics, and the original single-stranded region remained bound in the groove with minimal changes (Fig. S5). The “displaced” 5′ end overhang points toward the back of domain 3, which acts as a steric block for base pairing. This disposition is enforced by the extended conformation of the bound strand in which the bases are facing into the groove, whereas the backbone is steered by solvent-exposed positively charged residues at the rim. This configuration prevents reassociation of the two strands at the exit groove, which would happen if the complementary strand were to travel along the front of domains 1 and 2. This configuration also prevents the displaced strand to interfere with ATP-binding cleft dynamics.

### Probing the role of conserved RNA-binding residues in the RNA-stimulated ATPase activity

Using MD simulation, we have identified NS3H RNA-binding residues (Table 2). Based on their conservation among flaviviruses (Fig. S2*A*), we have designed several NS3H mutants having residues R231, T270, R274, D296, and K394 substituted with alanine (A). All mutants were produced as soluble proteins, and their ATPase activity and RNA-binding affinity were examined (Fig. S6 and Table 3). Mutations of T270A, D296A, and K394A caused only modest activity reduction (*k*_cat_ reduction), whereas R274A exhibited approximately fourfold reduction. The R231A mutant was almost inactive (∼20 fold reduction), preventing us from obtaining meaningful *K*_*m*_ values.

**Table 3:** Summary of kinetic constants for ATP substrate on ATPase activity and dissociation constants for RNA-binding affinity

Protein	Apparent *K*_*m*_ (μM)	Apparent *k*_cat_ (s^−1^)	*k*_cat_/*K*_*m*_ (s^−1^ μM^−1^)	RNA-binding affinity (*K*_*D*_, nM)
WT	125 ± 15	8.8 ± 0.2	0.07 ± 0.01	49.7 ± 9.3
R231A	ND	0.4 ± 0.2	—	>300
T270A	87 ± 13	6.9 ± 0.3	0.08 ± 0.02	86 ± 18
R274A	31.1 ± 6.8	2.3 ± 0.4	0.07 ± 0.06	141 ± 21
D296A	105 ± 13	7.3 ± 0.4	0.07 ± 0.03	29 ± 12
K394A	74.4 ± 9.1	5.2 ± 0.5	0.07 ± 0.05	118 ± 21

Abbreviation: ND, not determined.

Values are reported as average from repeated experiments (n ≥ 3) ± standard error of the mean.

As expected, mutations disrupting protein–RNA backbone contacts (T270A, R274A, K394A, and R231A) reduced RNA-binding affinity (Table 3). However, their contribution to binding is not equal. While the 2′-OH hydrogen bond with T270 hydroxyl contributes to the overall affinity only marginally, the salt bridge between R231 guanidinium group and backbone phosphate is pivotal. In contrast, the base-specific contact (D296) is reducing the overall affinity of the WT protein (as manifested by approximately twofold affinity increase for D296A), presumably because of considerable electrostatic repulsion between the side chain and phosphate backbone within the RNA-binding groove filled with bases of relatively low dielectric constant. Thus, in addition to recognizing a specific viral sequence, this residue may prevent stalling of the helicase during translocation because of too high affinity.

The backbone contact mutations simultaneously diminish catalytic turnover (*k*_cat_) and *K*_*m*_ leaving the catalytic efficiency (*k*_cat_/*K*_*m*_) unchanged (Table 3). This indicates that RNA backbone binding modulates both the rate-limiting step as well as nucleotide exchange and that multiple residues from both domains are involved. Plausible roles of the mutated residues are delineated below with the help of RNA–protein contacts identified in MD simulations (Table 2).

Repositioning of α7 helix and opening of the ATP-binding cleft (Fig. 5*A*) is associated with formation of modest salt bridge between K394 side chain and RNA backbone phosphate in the low-affinity state (Table 2). This contact is absent in the high-affinity state. Thus, this RNA–protein contact may couple opening and closing of the ATPase active site to stepping between or handover of backbone phosphates. On the other hand, R231 mediates strong and essential salt bridge between domain 1 and RNA. This conserved arginine is within motif Ia at the tip of α2 helix and is involved in RNA binding in other flaviviral helicases (30). Given its strong bond to RNA, which persists in the low-affinity state (Table 2), it is plausible that this contact acts as a sensor of RNA movement that is then transmitted to α2 helix and ultimately coupled to the rate-limiting step within the active site. R274 may track the RNA movement in similar fashion, albeit with lower affinity.

In contrast to the aforementioned salt bridges, removal of the T270 hydrogen bonding with ribose 2′-OH (Table 2) had only modest effect on affinity and activity. Similar contacts were previously identified in this conserved threonine residue (Fig. S2*A*) for DENV (11, 15) and ZIKV (17) helicase, and protein residue contacts with ribose 2′-OH were shown to be responsible for RNA/DNA discrimination for Mss116, a nonviral DEAD-box helicase that belongs to SF2 (31). Given the rather modest effect of T270A mutation, it is likely that this residue alone cannot be responsible for RNA specificity in flaviviruses and other, nonconserved residues, such as R536 in TBEV (Table 2), may contribute. In addition, more flexible ssDNA may incur higher entropic penalty when adopting the rather extended RNA backbone within the helicase groove (32).

In summary, conserved RNA-binding residues contribute to the binding but with varying affinity that may reflect their distinct roles in the helicase catalytic cycle.

### Structural basis of DNA inhibition

Biochemical data show that ssDNA acts as an inhibitor of RNA-stimulated ATPase activity. The inhibition is incomplete (Fig. 1*D*) and requires much higher ssDNA concentrations (micromolar range) than RNA binding (*K*_*D*_ ∼ 50 nM, Fig. 1*E*), thus suggesting that DNA does not directly compete with RNA for the binding cleft. This is further supported by only partial binding competition even at high DNA concentrations (anisotropy, Fig. 1*F*).

Furthermore, the increased effectiveness of longer ssDNA_41_ suggests that binding to multiple low-affinity surface sites might be involved. The electrostatic surface charge distribution (NS3H–RNA model, Fig. S7) suggests that even in the presence of RNA, there are positively charged patches around the ATP-binding site and near the exit site of the RNA groove on domain 1. Hence, DNA binding along the ATP-binding cleft might be a plausible way to interfere with the ATPase cycle, for example, by biasing the conformation of the ATPase site toward inactive configuration or modulating the ATP affinity through active-site occlusion.

**Figure 6:**
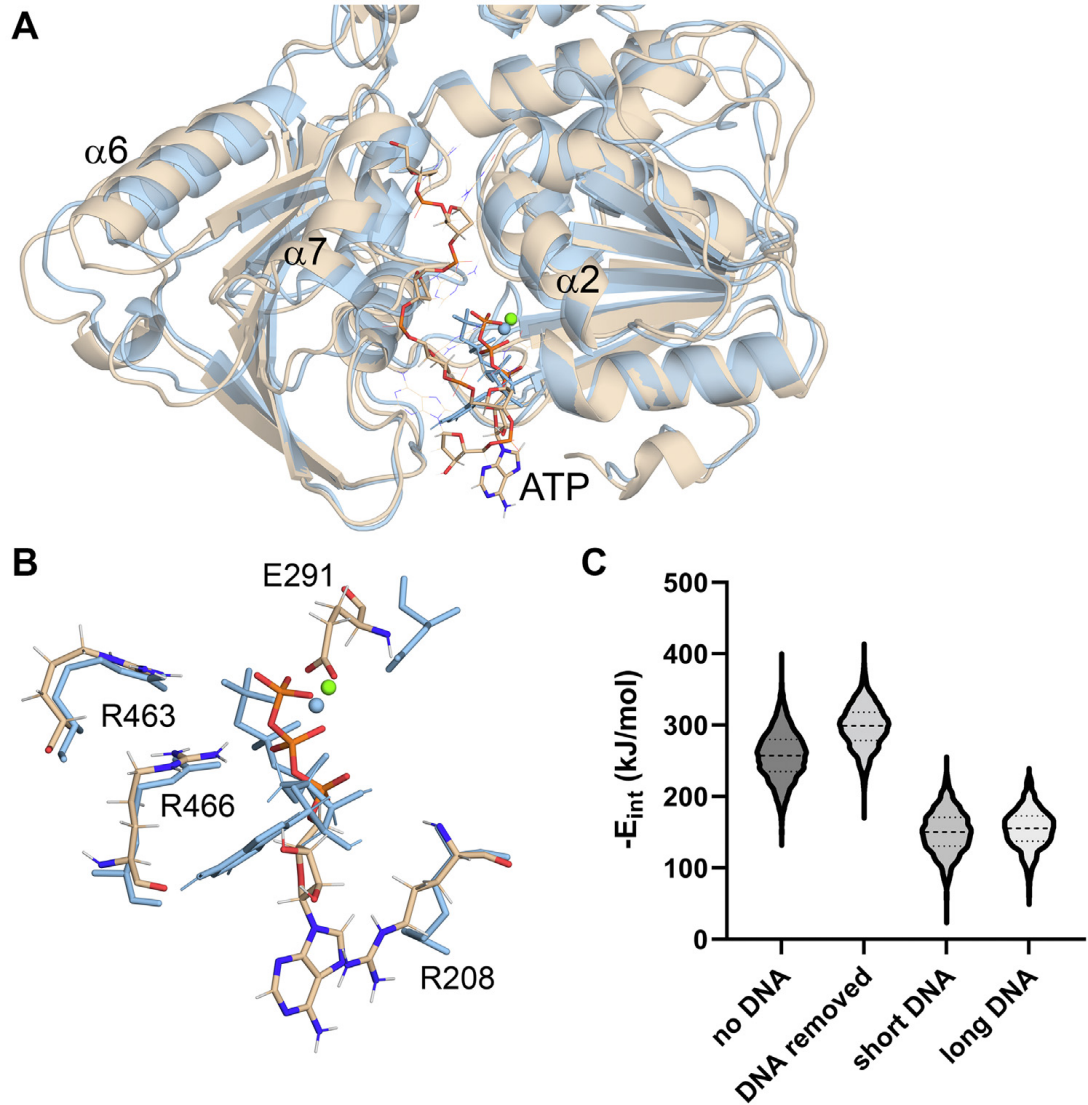
**Effects of short ssDNA association on ATP binding.***A*, the conformational changes upon DNA binding (final conformation, bases in wireframe, and backbone in *stick* representation), Initial ATP state is shown in *cyan sticks*, the final state in *sticks* reflecting atomic composition (carbon atoms—*wheat*, hydrogen—*light gray*, oxygen—*red*, nitrogen—*blue*, and phosphorus—*orange*. The protein backbone is shown as a *ribbon* (*final state wheat*). The *green sphere* indicates the position of Mg in final state. *B*, close-up of the ATPase active site showing γ-phosphate displacement. *C*, change in ATP affinity upon DNA binding measured by GBSA interaction energy (−*E*_int_) distributions shown as violin plot. Short DNA refers to hexanucleotide sequence 5′-AGACTA-3’. GBSA, generalized Born surface area.

We have explored this hypothesis by constructing a model based on the NS3H–AMPPNP structure (with AMPPNP replaced by ATP) and adding a short (hexanucleotide) DNA with B-form backbone running along the ATP-binding cleft. The initial model was subjected to MD and rapidly converged to a stable ensemble of configurations (Fig. 6*A*). As controls, two MD simulations were done: one without the bound DNA and another starting from the last frame of the NS3H–ATP–DNA complex but with the bound DNA removed. Analysis of ATP interaction energy distributions obtained from the stationary portions of these stimulation trajectories (as judged by RMSD, Fig. S8*A*) revealed that DNA binding along the ATPase active site reversibly decreases the ATP interaction energy by about twofold (Fig. 6 *C*). A similar effect is observed when ssDNA_41_ is placed across the cleft (Fig. 6*C*). In this case, the DNA, which in the initial model, is represented by a long straight B-type helix, folds and bends, and interacts with multiple positively charged patches (*cf.*, surface charge map in Fig. S7). While binding along the ATPase cleft seems persistent, the interactions with positive patches II and III (Fig. S7) are transient on the nanosecond time scale (Movie S2).

Given that ATP binding is dominated by polar and electrostatic interactions of the triphosphate moiety and the adenine base is highly mobile (as seen in MD simulations), it is not surprising that the DNA-induced decrease in interaction energy is associated with displacement of the triphosphate moiety within the ATPase active site (Figs. 6*B* and S8*B*). Such displacement would be enough to disrupt the precise alignment of the catalytic residues, water, and the γ-phosphate and may lead to inhibition of the ATPase cycle. DNA binding may also interfere with nucleotide exchange by sterically blocking access to the ATPase site, and the longer DNA also partially occludes the RNA-binding groove, explaining the observed partial and weak competition between the two nucleic acids.

We compared the effect of DNA to another polyanion heparin, which is often used to compete with nucleic acid binding. Unlike ssDNA, heparin can effectively and fully inhibit RNA-stimulated ATPase activity at comparable micromolar concentrations (of charged units, *e.g.*, nucleotide or sulphated sugar) likely *via* direct competition with RNA (Fig. S9, *A*–*C*). Similarly, near complete inhibition of the ATPase activity can be achieved by high salt concentration, highlighting the prominent role of electrostatic interactions in RNA and ATP binding (Fig. S9*D*).

In contrast, significant but still incomplete ATPase inhibition could only be achieved at high DNA concentrations (250 μM and above, corresponding to ∼10 mM in phosphate group equivalent, Fig. 1*D*). In comparison, full ATPase inhibition is achieved at 10 μM heparin, which corresponds to 30 μM negative sulfate charge. This indicates that heparin effectively competes with RNA for the binding site, whereas ssDNA fails to do so and must exert its effect by a different mechanism. This is consistent with the much lower binding affinity of the short ssDNA (apparent *K*_*D*_ >15 μM, Fig. S9*E*) when compared with that of the equivalent RNA oligomer (*K*_*D*_ ∼ 50 nM, Fig. 1*E*). Partial competition with RNA is only seen for longer ssDNA suggesting that ssDNA exploits multiple weaker binding sites, which might augment the overall affinity and either partly occlude the RNA-binding groove (as seen in Fig. S8*B*) or even force a portion of ssDNA to adopt otherwise unfavorable extended conformation within the RNA-binding site. The latter would be consistent with partial activation of the ATPase at high concentration of long DNA (Fig. 1*A*, ssDNA_41_). The different mechanism of inhibition by heparin and ssDNA, respectively, is likely because of their different polymer behavior. Heparin, with its high charge density, is likely to prefer extended configurations in a fashion similar to the stiffer RNA (longer persistence length than ssDNA (32)) and thus fit into the RNA-binding groove. In contrast, the softer ssDNA entropically “resists” such constrained extended conformations and instead prefers nonspecific binding to multiple charged surface patches employing many configurations. Thus, ssDNA inhibition relies on polyanion nature of the polymer, which tethers distant binding patches and indirectly exerts allosteric changes to the ATPase site. While this effect is unlikely to play any role in the replication cycle within cytoplasm, this mechanism might provide lead principle for development of novel non-nucleotide allosteric inhibitors.

## Conclusion

Current study provides a structural and mechanistic insight into the ATP hydrolysis cycle of TBEV helicase. Several conformational changes associated with individual ATP hydrolysis steps have been captured in crystallographic structures of NS3H–AMPPNP–Mn^2+^, -ADP–Pi–Mn^2+^, -ADP–Mn^2+^, and the apo form of the protein. The overall structure of TBEV helicase is highly similar to that of other flaviviruses such as DENV and ZIKV, suggesting similar ATP hydrolysis and RNA translocation mechanism. However, contrary to the mechanism proposed for DENV helicase (9), our structural and biochemical data are consistent with Pi release being the rate-limiting step of the ATPase, as also seen for the HCV NS3 helicase (19). By mutating several conserved RNA-binding residues, we identified three residues (R231, R274, and K394) involved in allosteric communication between the RNA-binding groove and the ATPase site. Despite forming structurally similar salt bridges with the phosphate backbone, these residues contribute differently to RNA-binding energetics and are likely to play distinct roles in the coupling between RNA translocation and ATPase cycle.

Furthermore, we found that ssDNA inhibits RNA-stimulated ATPase activity but not by directly competing with RNA for the oligonucleotide-binding cleft. Using modeling and MD simulations, we propose a plausible explanation in which ssDNA exploits nonspecific binding to positively charged surface patches in the vicinity of the ATP-binding pocket, allosterically weakening ATP interactions, and consequently leading to the repositioning of the triphosphate moiety. However, this is just one plausible explanation, and the binding of longer DNAs might have additional effects on protein dynamics, for example, preventing conformational changes that are necessary for phosphate release or nucleotide exchange. While ssDNA is irrelevant to the virus replication cycle in the cytoplasm where it is unlikely to encounter this type of nucleic acid, it might open new ways to design polyanionic allosteric inhibitors that combine this “parasitic” mode with more specific targeting.

## Experimental procedures

### Plasmid construction and protein production

A DNA fragment encoding the full-length helicase domain, including the linker region between protease and helicase domains (amino acid residues 173–621) from TBEV strain HYPR, hereafter referred as NS3H, was amplified from pUC57-HYPR-FRAGII (kindly provided by Prof Daniel Růžek, Masaryk University, Czech Republic) using Q5 polymerase and gene-specific primers (Table S1). The PCR product was cloned into pET-19b plasmid (Novagen) and sequence verified. Point mutations affecting RNA binding (R231A, T270A, R274A, D296A, and K394) were introduced using the Q5 Site-Directed Mutagenesis Kit (New England Biolabs) and corresponding primers (Table S1). The presence of the mutations was confirmed by sequencing.

Recombinant NS3H proteins (wildtype and mutants) were produced in *Escherichia coli* BL21-CodonPlus (DE3)-RIPL competent cells (Agilent Technologies). Briefly, transformed competent cells were grown at 37 °C in LB media supplemented with 100 μg/ml ampicillin and 35 μg/ml chloramphenicol until *A*_600nm_ reached 0.5. The cells were chilled for 30 min at 4 °C, and the protein production was induced with 1 mM IPTG followed by incubation for 20 h at 18 °C or 5 h at 30 °C. The cells were harvested by centrifugation at 4000*g* for 30 min at 4 °C and stored at −80 °C prior to further use.

### Protein purification

Cells were resuspended in buffer A (0.02 M sodium Hepes, pH 7.0, 0.5 M NaCl, and 10 μg/ml DNAse I) and lysed using LM20 Microfluidizer Processor (Microfluidics). The resulting cell lysate was clarified by ultracentrifugation at 40,000*g* for 60 min at 4 °C, and collected supernatant was loaded on HisTrap FF column (Cytiva) equilibrated in buffer A. Histidine-tagged NS3H was eluted using 70% of buffer B (0.02 M sodium Hepes, pH 7.0, 0.5 M NaCl, and 1 M imidazole). Fractions containing recombinant NS3H were pooled together, and the elution buffer was exchanged for buffer C (0.02 M sodium Hepes, pH 7.0, and 0.15 M NaCl) using Amicon Ultra-15 spin columns with 30 kDa cutoff (Merck). To remove the residual nucleic acids bound to NS3H, the sample was loaded into HiTrap Heparin HP column (Cytiva) pre-equilibrated in buffer C, and NS3H protein was eluted using 1 M NaCl. Finally, eluted NS3H was concentrated using Amicon Ultra-15 with 30 kDa cutoff spin columns and loaded into a Superdex 200 Increase 10/300 GL (Cytiva) pre-equilibrated in buffer C for final polishing. The purity of the protein was analyzed using an SDS-PAGE gel and MALDI-TOF mass spectrometry.

### Crystallization, data collection, and data processing

The apoNS3H crystal was obtained using a sitting drop vapor diffusion method in 96-well plates assisted by Oryx-6 crystallization robot (Douglas Instruments) at 18 °C. The protein in buffer C (2.2 mg/ml) was crystallized using Ligand-Friendly Screening HT-96 (Molecular Dimensions) for the initial screening, and protein crystals grew in 0.1 M Bis–Tris propane, pH 6.5, 0.2 M sodium acetate trihydrate, 20% w/v PEG 3350, and 10% v/v ethylene glycol. Crystals for NS3H–ADP–Mn^2+^ complex were obtained by cocrystallization of NS3H (2.2 mg/ml) with 5 mM MnCl_2_ and 5 mM ADP (Sigma–Aldrich) in 0.1 M Bis–Tris propane, pH 6.5, 0.2 M sodium acetate trihydrate, 20% w/v PEG 3350, and 10% v/v ethylene glycol. Crystals for NS3H–AMPPNP–Mn^2+^ complex were obtained by cocrystallization of NS3H (2.2 mg/ml) with 5 mM MnCl_2_ and 5 mM AMPPNP (Sigma–Aldrich) in 0.1 M Bis–Tris propane, pH 6.5, 0.02 M sodium–potassium phosphate, pH 7.5, 20% w/v PEG 3350, and 10% v/v ethylene glycol. With the same condition, NS3H was cocrystallized with 5 mM ATP (Thermo Fisher Scientific) and 5 mM MnCl_2_. For data collection, every single crystal was fished out and flash cooled in liquid nitrogen without any additional cryoprotection. X-ray diffraction intensities were collected on BL14.1 at BESSY II electron-storage ring operated by the Helmholtz–Zentrum Berlin (33). The collected datasets were processed using *XDS* (34) with the *XDSAPP* graphical user interface (35). Data collection and processing statistics are given in Table 1.

### Structure determination and refinement

The apoNS3H structure was solved by a molecular replacement with the program *MOLREP* (36) from the *CCP4i2* suite (37) using ZIKV helicase (PDB code: 6ADW) as a starting model. The ADP–Mn^2+^ and AMPPNP–Mn^2+^ ternary complexes were solved using the apoNS3H structure as the starting model. Where applicable, translation/libration/screw (TLS) groups were initially identified using TLS motion determination (38) followed by restrained and TLS refinement cycles using REFMAC5 (39) and interspersed with manual model rebuilding using the *Coot* software (GNU General Public License) (40). The quality of the structure was analyzed using *MolProbity* (GNU General Public License) (41). Refinement statistics and stereochemistry analysis are shown in Table 1. Superposition of structures and figures were prepared using PyMOL (Schrödinger, Inc) (42) and UCSF Chimera (RBVI, University of California, San Francisco) (43).

### ATPase assay

ATPase activity of NS3H was monitored using EnzChek Phosphate Assay Kit (Molecular Probes, Inc), enabling the quantification of Pi release, which is translated as the rate of ATP hydrolysis by NS3H. The assay was done in a final volume of 200 μl in a 96-well plate according to the manufacturer's instructions. Briefly, 6.25 nM NS3H was mixed with individual assay components in a reaction buffer supplied by the kit and with either RNA (poly(A), 1.4 mM; Merck) or ssDNA_41_ (1 mM; Table S1) and incubated for 10 min at 30 °C. The hydrolysis reaction was started by adding ATP (Thermo Fisher Scientific). Steady-state kinetics of Pi release from ATP hydrolysis was measured using a Synergy H1 microplate reader (BioTek Instruments) at 360 nm. For determination of kinetics constant of ATP substrate, the initial velocity of the reaction was calculated and expressed in micromolar concentration of Pi released per second interpolated from a calibration curve done using KH_2_PO_4_ standard solution. Data were fitted to the Michaelis–Menten equation using GraphPad Prism 9.0 (GraphPad Software, Inc) as follows:

(i) $$v=\frac{V_{\text{max}}\cdot \left[S\right]}{K_{m}+\left[S\right]}$$

(ii) $$v=\frac{Et\;\cdot k_{\text{cat}}\cdot \left[S\right]}{K_{m}+\left[S\right]}$$

where *V*_max_ is the maximum velocity at given enzyme concentration, *K*_*m*_ is the Michaelis–Menten constant, *Et* is the molar enzyme concentration (measured by UV absorption at 280 nm using ε_M_ = 78,295 M^−1^ cm^−1^, estimated by ProtParams utility; https://web.expasy.org/protparam/), *k*_cat_ is the turnover number, and *S* is the concentration of ATP substrate.

### Fluorescence anisotropy–based binding assay

Fluorescence anisotropy–based binding assay was performed by adding 10 nM 6-FAM-ssRNA_12_ (Table S1) into serial dilutions of NS3H in an assay buffer (20 mM Tris–HCl, pH 7.5, 100 mM NaCl, 1 mM MgCl_2_, and 7.5% glycerol). The reaction mixtures (100 μl) in triplicates were incubated for 45 min at 30 °C prior to measurements in Thermo Fisher Scientific black 96-well immunoplates using Synergy H1 microplate reader. Anisotropy (*r*) values were calculated as follows:

(iii) $$r=\frac{\left(I_{\left|\right|}-I_{⊥}\right)}{\left(I_{\left|\right|}+2I_{⊥}\right)}$$

where $I_{\left|\right|}$ is the parallel emission signal and $I_{⊥}$ is the perpendicular emission signal. Observed anisotropy values were plotted as a function of protein concentration. Dissociation constant (*K*_*D*_) between NS3H and RNA was determined by nonlinear curve fitting of fluorescence anisotropy data to (Equation iv) using GraphPad Prism 9.0.

(iv) $$r=r_{\text{min}}+\frac{\left(r_{\text{max}}-r_{\text{min}}\right)}{2\left[L\right]}\left(\left(K_{D}+\left[R\right]+\left[L\right]\right)-{\left[{\left(K_{D}+\left[R\right]+\left[L\right]\right)}^{2}-4\left[R\right]\left[L\right]\right]}^{1/2}\right)$$

where *r*, *r*_min_, and *r*_max_ are the observed, minimum, and maximum anisotropy, respectively, and [*R*] and [*L*] is the concentration of NS3H and 6-FAM-ssRNA_12_, respectively.

Effect of DNA on NS3H–RNA interaction was examined by adding 800 nM unlabeled-ssDNA_12_, -ssDNA_20_, or -ssDNA_41_ (Table S1) into a mixture of 10 nM 6-FAM-ssRNA_12_ and 100 nM NS3H in the assay buffer.

### MD simulation

All simulations were performed using GROMACS (GNU General Public License) (44) with AMBER99SB-ILDN force field (26) for proteins, nucleic acids, and ions. Simulations were performed at a constant temperature of 300 K (maintained by a modified Berendsen thermostat (45)) and pressure of 1 bar (Parrinello–Rahman coupling (46)). A generalized AMBER force field (47) employing the standard procedure (48) was used for ATP. The complexes were solvated in an octagonal box using the TIP3P explicit water model. Chloride and/or sodium ions and Mg^2+^ (in the presence of ATP) were added to neutralize the overall net charge. All simulations were performed for a minimum of 0.5 μs or longer until stable configuration was achieved (verified by stable RMSD for over 100 ns). ApoNS3H structure (this work, PDB code: 7OJ4) was used, and residues missing from the density maps (*i.e.*, flexible loops) were modeled using PyMOL (42). Short (hexamer) RNA oligomers encompassing part of the conserved 5′-UTR from the TBEV genome (5′-AGAUUU-3′) was modeled using the resolved oligonucleotide (5′-AGACUA-3′) in the crystal structure of DENV4 NS4h–RNA_12_ complex (PDB code: 2JLU) (11) and protein structure superposition. For the simulation of partially double-stranded substrate, the MD equilibrated structure with the aforementioned single-stranded oligo was replaced by sequence AAACUA, which eliminates base-specific contacts, and extended at the 5′ and 3′ ends to 5′GCCAAAAAACUAUUUU and base-paired at the 5′ end with a partially complementary (6 base pairs) strand 5′ AAAAUUUGGC, which represents a split fork with potentially weaker A–U base pairs at the junction. The system was simulated as described previously.

Models of heterologous nonspecific surface binding were constructed using ssDNA_6_ or long ssDNA_41_ (Table S1) using initial B-form backbone and base stacking conformation. The helix was placed in the vicinity at various places around the surface of RecA domains and then simulated. Simulations with the ATP placed in the binding site (using the position of AMPPNP in the crystal structure PDB code: 7BM0 for initial placement) were also performed to explore the effects of DNA on ATP binding. Relative binding energies were computed for subset of the simulation frames using the generalized Born surface area method with inclusion of the Still’s model (49) for computation of the effective Born radii implemented in the GROMACS program package (44). Superposition of structures and figures was prepared using UCSF Chimera (43).

## Data availability

The atomic coordinates and experimental structure factors were deposited in the PDB under accession codes: 7OJ4 (apoNS3H), 7BLV (NS3H–ADP–Mn^2+^), 7BM0 (NS3H–AMPPNP–Mn^2+^), and 7NXU (NS3H–ADP–Pi–Mn^2+^). All remaining data are present within this article.

## Supporting information

This article contains supporting information (50, 51).

## Conflict of interest

The authors declare that they have no conflicts of interest with the contents of this article.
