# Supplementary material for: Mechanistic insight into the RNA-stimulated ATPase activity of tick-borne encephalitis virus helicase
Source: J Biol Chem. 2022 Aug 17;298(10):102383. doi: 10.1016/j.jbc.2022.102383 (PMC9490040; doi:10.1016/j.jbc.2022.102383)
Supplement: Supporting Information [file mmc3.pdf]

Supporting Information

Mechanistic insight into the RNA-stimulated ATPase activity of tick-borne encephalitis virus helicase

Paulina Duhita Anindita, Marco Halbeisen, David Řeha, Roman Tuma, Zdenek Franta*

Department of Chemistry, University of South Bohemia in České Budějovice, Czech Republic

*Corresponding author: Zdenek Franta
E-mail: zfranta@prf.jcu.cz

Contents:

Table S1. List of oligonucleotides used in the study.
Figure S1. Purification of recombinant NS3H.
Figure S2. Structural-based sequence alignment and residue conservation of NS3 helicases.
Figure S3. Comparison between α7 and AMPPNP molecule position from AMPPNP-Mn^{2+}-bound NS3H (PDB: 7BM0) and DENV helicase (PDB: 2JLR).
Figure S4. MD simulation.
Figure S5. MD simulation involving NS3H and dsRNA.
Figure S6. ATPase activity and RNA binding affinity of NS3H mutants.
Figure S7. Surface electrostatic energy distribution for NS3H:RNA complex model.
Figure S8. MD simulation involving NS3H and DNA.
Figure S9. Effect of salt and heparin on NS3H ATPase activity and NS3H-DNA binding.

Table S1. List of oligonucleotides used in the study

**Primer for cloning into pET-19b vector**	
NS3Hel_NdeI_F[a]	5'-TGCTAGCATATGGAGAAGAGTCGACCCAACCTCCC-3'
NS3Hel_XhoI_R[a]	5'-TGCTAGCTCGAGTTAGCGACGCCCAGATGCGTA-3'
NS3H_R231A_F	5'-GGCTCCAACTGCTGTGGTACTCAAAG-3'
NS3H_R231A_R	5'-AACACCAATGTTCTCAGG-3'
NS3H_T270A_F	5'-GTGTCACGCAGCTTATGTCAACAG-3'
NS3H_T270A_R	5'-ATCACATCGACAATTGCC-3'
NS3H_R274A_F	5'-CTATGTCAACGCTCGGCTATTACCACAG-3'
NS3H_R274A_R	5'-GTTGCGTGACACATCACA-3'
NS3H_D296A_F	5'-CCACTGGACTGCTCCTCACAGCATA-3'
NS3H_D296A_R	5'-GCCTCATCCATGATTGCC-3'
NS3H_K394A_F	5'-TTTGAACAGCGCTACCTTTGAAAAGGAC-3'
NS3H_K394A_R	5'-CAGATCACACTTTTCCCTTTC-3'
**Labeled ssRNA and ssDNA for fluorescence anisotropy binding assay**	
6-FAM-ssRNA$_{12}$	[6-FAM]-5'-AGAUUUUCUUGC-3‘
6-FAM-ssDNA$_{12}$	[6-FAM]-5'-AGATTTTCTTGC-3‘
**Unlabeled ssDNA for ATPase/fluorescence anisotropy binding assays/MD simulation**	
ssDNA$_{6}$	5'-AGACTA-3’
ssDNA$_{12}$	5'-AGATTTTCTTGC-3'
ssDNA$_{20}$	5'-AGAGGATCCCCGGGTACCGA-3'
ssDNA$_{41}$	5'-AGAGGATCCCCGGGTACCGATTAGATTATTGAGCTCTCCAG-3'

[a] Restriction enzyme sequence is underlined.

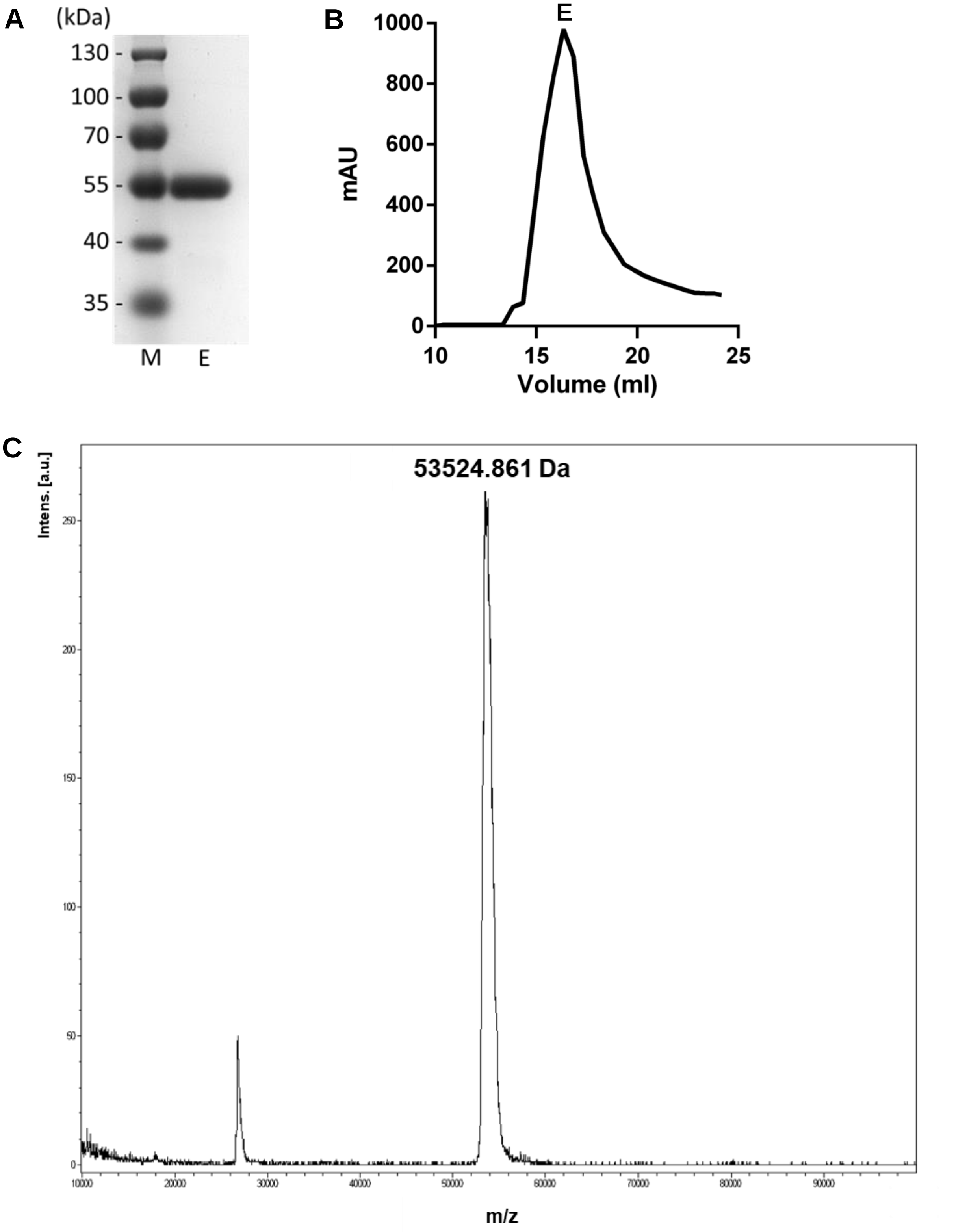

Figure S1. Purification of recombinant NS3H. (A) An SDS-PAGE analysis of eluted recombinant protein after gel filtration chromatography. Lane M is the SDS-PAGE molecular weight standard and lane E indicates the elution peak from gel filtration chromatography. (B) Chromatogram from gel filtration. Letter E indicates the elution peak correspond to panel (A). (C) MALDI-TOF spectrum of purified monomeric NS3H (~53 kDa), confirming the purity and size of recombinant NS3H. Note that the peak at m/z ~27,5 kDa represents a +2 charge species.

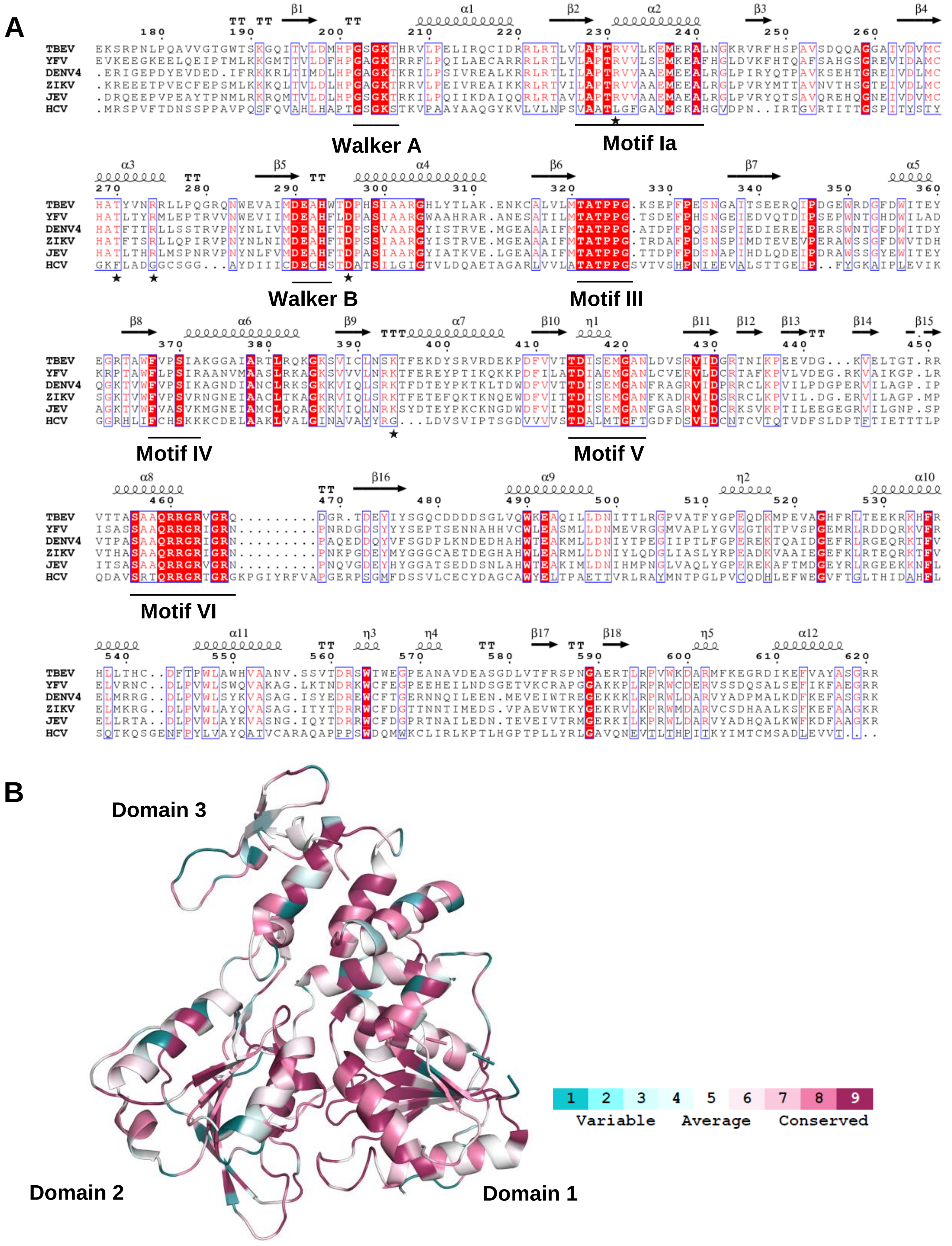

Figure S2. Structural-based sequence alignment and residue conservation of NS3 helicases . (A) Alignment of helicase domain from flaviviruses (TBEV, YFV, DENV4, ZIKV and JEV) and HCV generated using ESPript 3.0 (1). Conserved residues are highlighted in red while conserved SF2 helicase motifs are underlined and labeled. The conserved RNA binding residues mutated within the study are marked by black star.(B) Cartoon representation of NS3H residue conservation as calculated by the Consurf server (2) using the alignment in (A). The degree of residue conservation (variable, average and conserved) is color-coded as depicted in the figure.

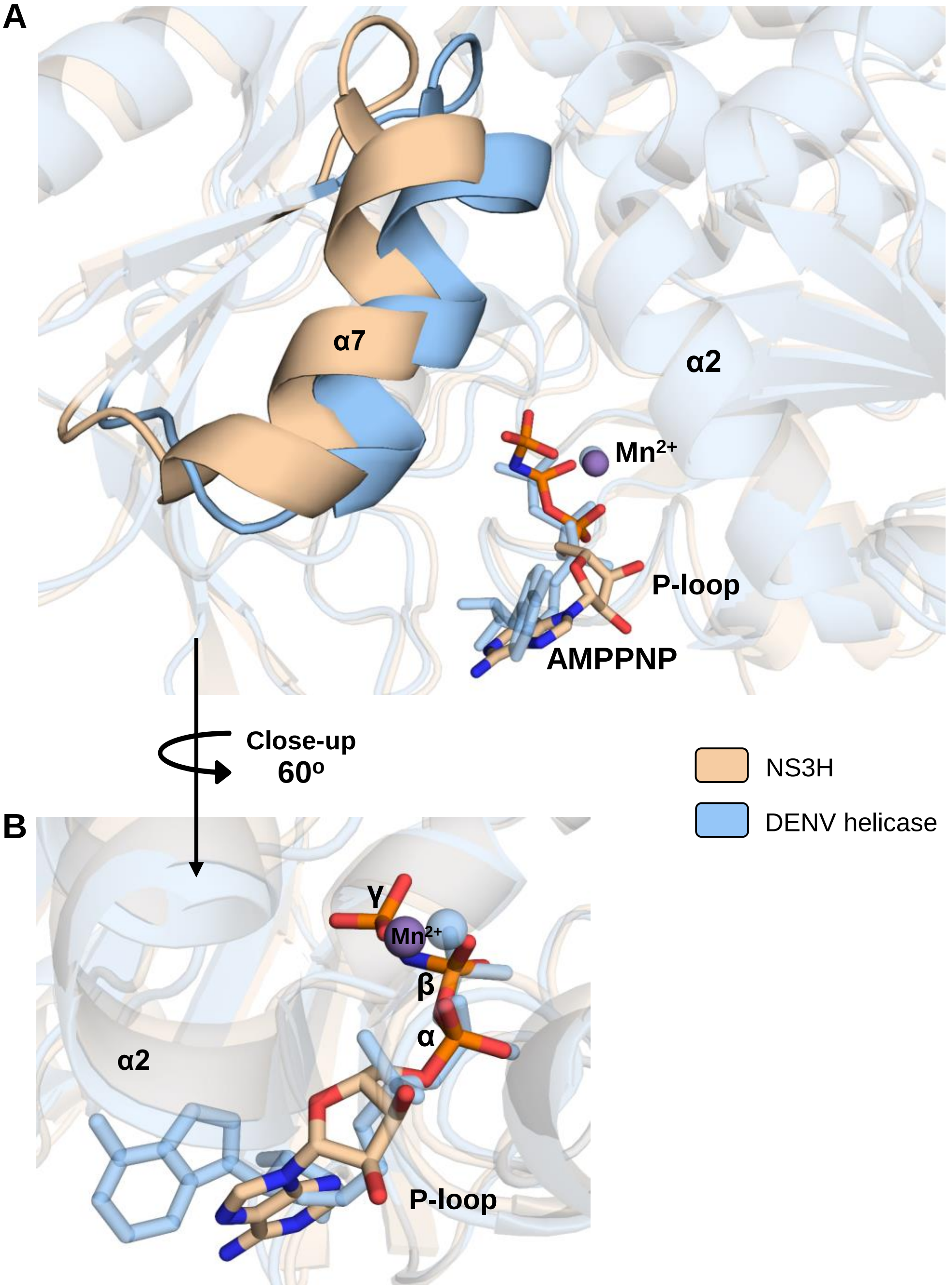

Figure S3. Comparison between α7 and AMPPNP molecule position from AMPPNP-Mn^{2+}-bound NS3H (PDB: 7BM0) and DENV helicase (PDB: 2JLR). (A) Superposition of α2, α7, and P-loop conformations from NS3H and DENV helicase. (B) Close-up view of ATPase site highlighting different position of AMPPNP molecule in NS3H and DENV helicase. Manganese ions are shown as purple (NS3H) and blue (DENV helicase) sphere.

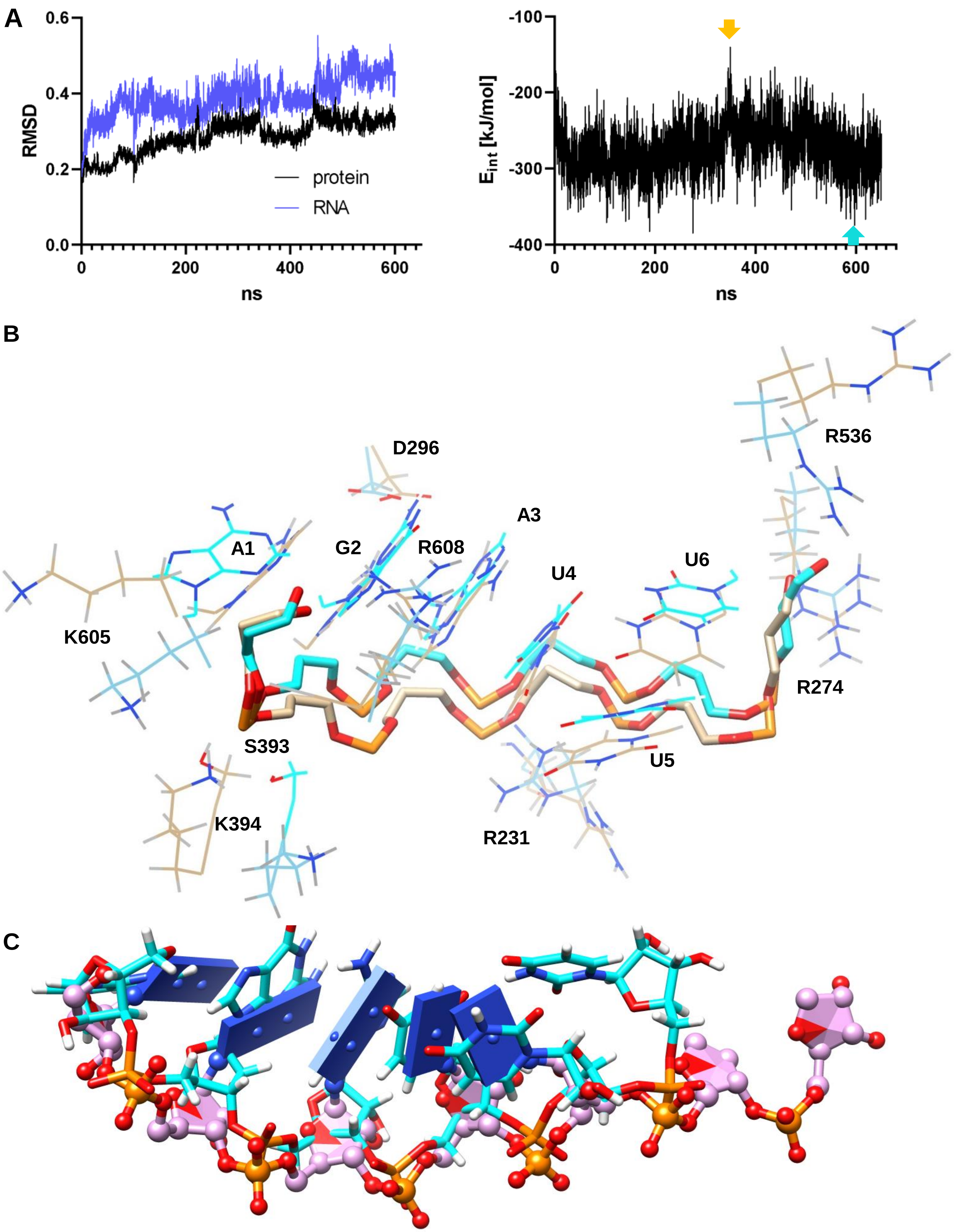

Figure S4. MD simulation. (A) Frame RMSD (left panel) and corresponding RNA interaction energy (GBSA approximation, right panel). (B) High (cyan) and low (wheat) affinity RNA-protein interaction comparison. RNA backbone in sticks, bases and protein side chains in wireframe. (C) Comparison between RNA configuration in DENV (PDB: 2JLU, in magenta and ball-and-stick representation) and high (cyan, sticks) affinity RNA configuration.

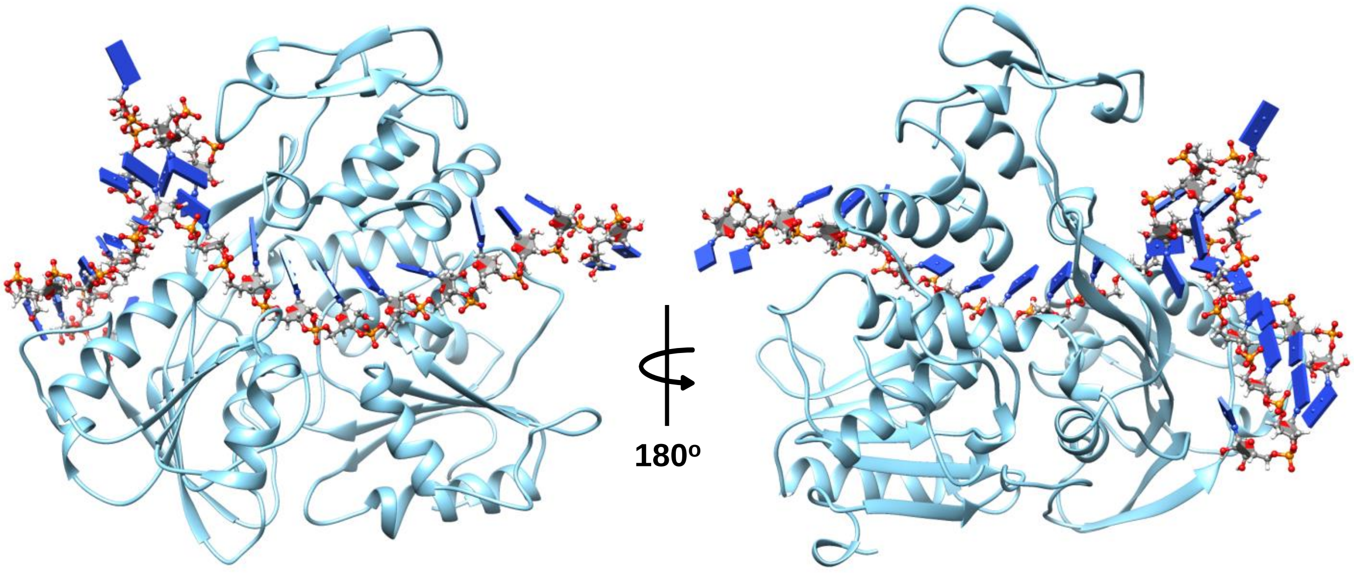

Figure S5. MD simulation involving NS3H and dsRNA. The front (left panel) and back (right panel) view of NS3H-dsRNA model are shown in cartoon (cyan) for NS3H and ball-and-stick for dsRNA.

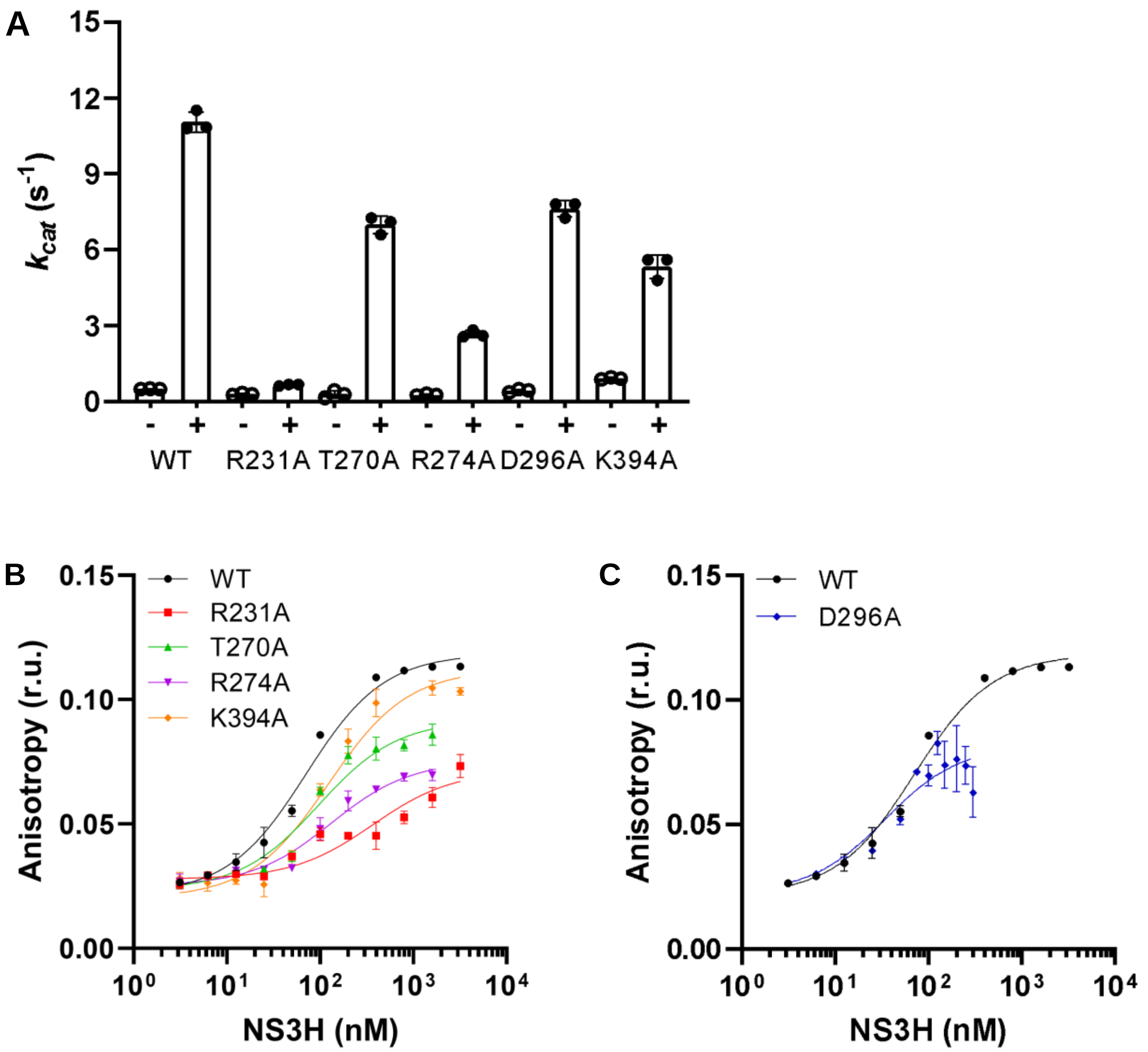

Figure S6. ATPase activity and RNA binding affinity of NS3H mutants. (A) ATPase activity of NS3H mutants measured using the ATPase assay as described in the "Experimental procedures" in the absence (-) and presence (+) of 1.4 mM poly(A). (B-C) NS3H-RNA binding was measured using fluorescence anisotropy using 10 nM 6-FAM-ssRNA$_{12}$ and increasing protein concentration from 3.1 nM to 3.2 µM for mutants R231A, T270A, R274A and K394A (B), and from 6.25 – 300 nM for mutant D296A (C). WT NS3H is shown for comparison. The amount of D296A protein used in the assay was lower than the rest of the mutants since the protein was most likely aggregating seen with the drop in the anisotropy at 300 nM D296A and larger standard deviation at protein concentration beyond 100 nM. Data are plotted as mean ± S.D., n=3.

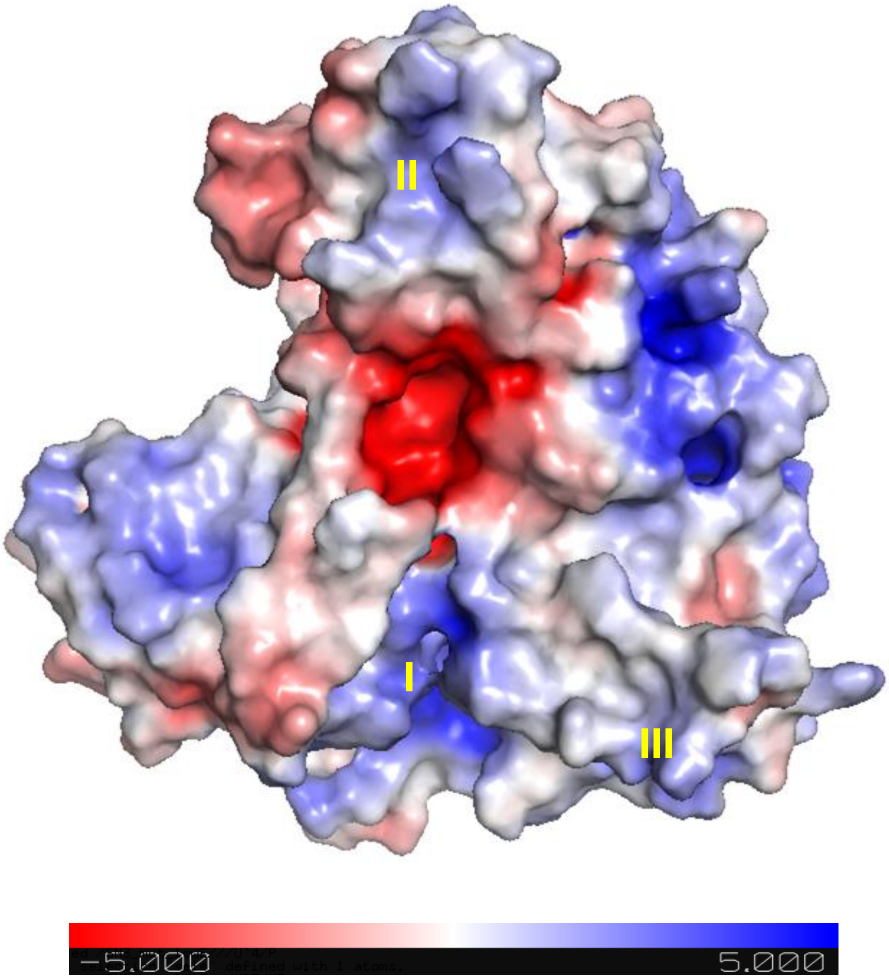

Figure S7. Surface electrostatic energy distribution for NS3H:RNA complex model. Positively charged surface areas that might bind additional nucleic acid are numbered (I-III) in yellow.

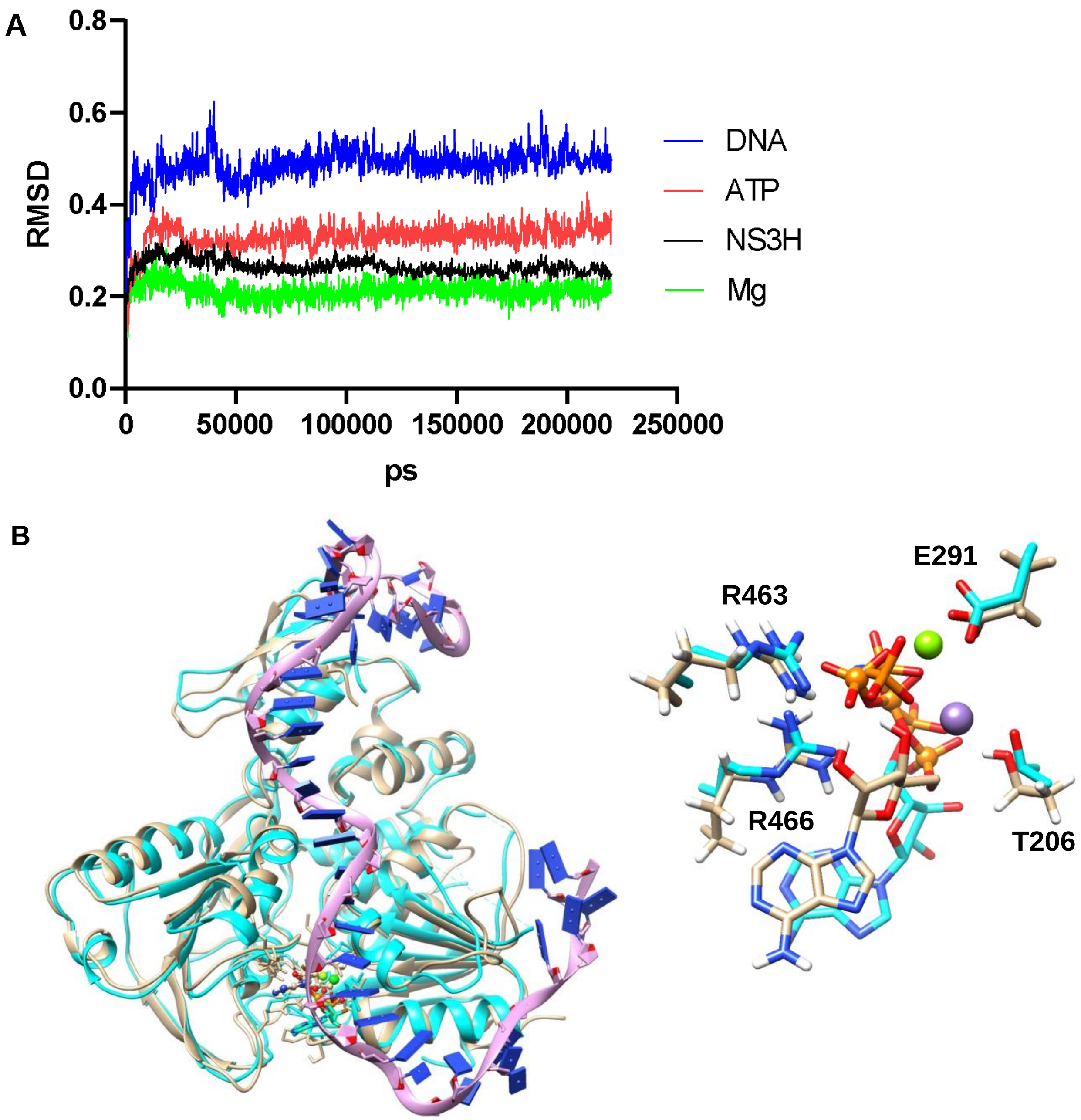

Figure S8. MD simulation involving NS3H and DNA. (A) RMSD of NS3H:ATP:Mg complex with short (hexamer) DNA bound along the ATPase site. (B) Left panel: A representative frame from simulation of $ssDNA_{41}$ (magenta backbone, blue bases) associated with NS3H:ATP:Mg complex. The initial state, NS3H:AMP-PNP:Mn crystal structure, is depicted in cyan while the final simulation frame is depicted in wheat. Right panel: Detail of ATP binding upon $ssDNA_{41}$ binding (initial state is cyan, final state is wheat, triphosphate in final state is depicted as ball & sticks). Mn atom is depicted as purple sphere and was replaced by Mg for simulation (green sphere, final state).

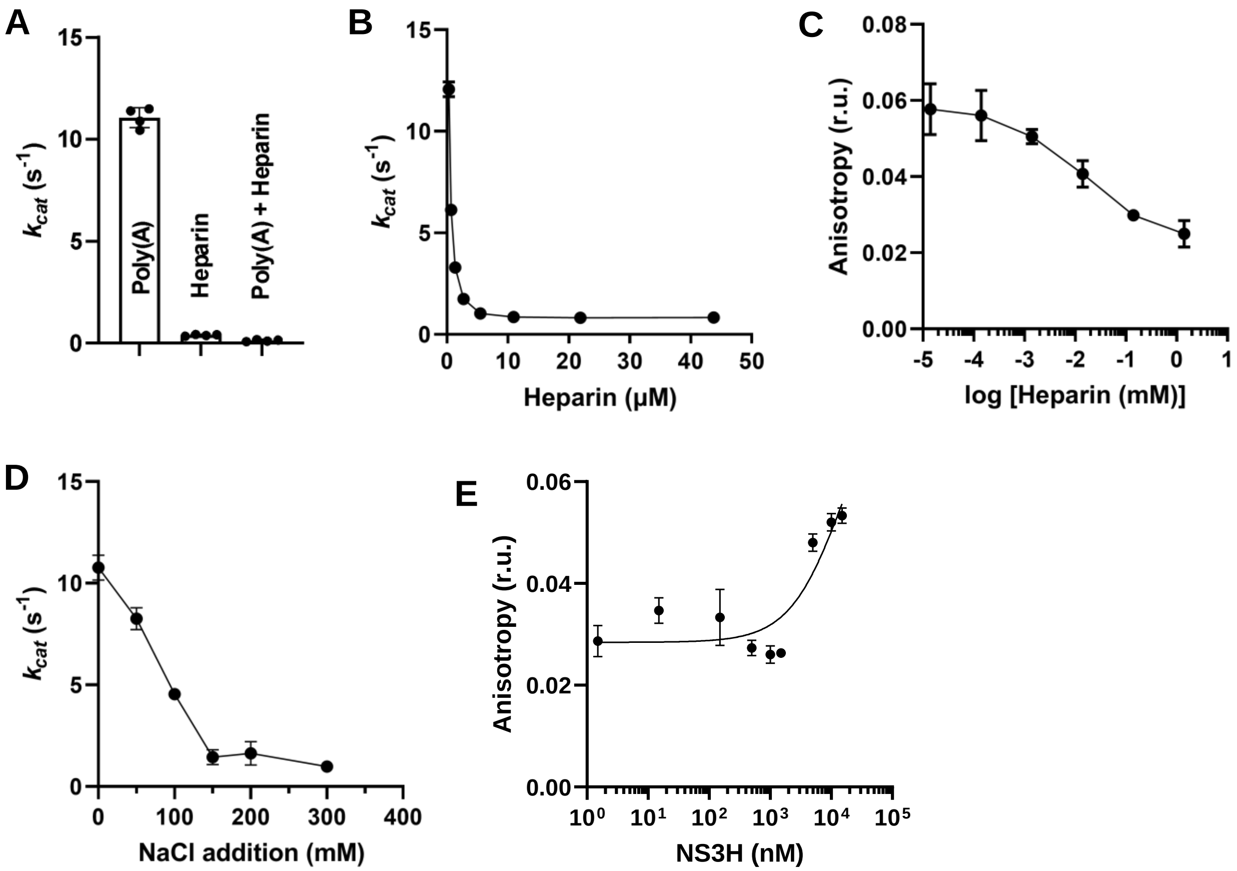

Figure S9. Effect of salt and heparin on NS3H ATPase activity and NS3H-DNA binding. (A) Heparin (at concentration 1 mM, measured as concentration of a single heparin subunit encompassing three negatively charged sulfate groups; PubChem CID: 44336410; effective mass = 623.5 Da) potently inhibited poly(A)-stimulated NS3H ATPase activity while heparin alone did not stimulate any ATPase activity as measured using an ATPase assay described in the "Experimental procedures" section. (B) Heparin inhibited ATPase activity stimulation by poly(A) in a concentration-dependent manner. Heparin was titrated by 2-fold serial dilution (14 nM – 45 µM) in the presence of 1 mM ATP and 1.4 mM poly(A) (mean ± S.D.). (C) Heparin competed with 6-FAM-ssRNA$_{12}$ as observed using fluorescence anisotropy in a concentration-dependent manner (mean ± S.D., n=3). (D) ATPase activity of NS3H was measured using an ATPase assay as described in the "Experimental procedures" section in different salt (NaCl) concentration. The initial salt concentration was 15 mM. NS3H ATPase activity in the presence of increasing salt concentration was measured individually and plotted as mean ± S.D., n = 3. (E) Binding of NS3H to 10 nM 6-FAM-ssDNA$_{12}$ as measured using fluorescence anisotropy binding assay (see "Experimental procedures") using increasing concentration of NS3H from 1.5 nM to 15 µM (mean ± S.D., n = 3).

References

(1) Robert, X. and Gouet, P. (2014) "Deciphering key features in protein structures with the new ENDscript server". *Nucl. Acids Res.* 42(W1), W320-W324.
(2) Ashkenazy H., Abadi S., Martz E., Chay O., Mayrose I., Pupko T., and Ben-Tal N. 2016 ConSurf 2016: an improved methodology to estimate and visualize evolutionary conservation in macromolecules. *Nucl. Acids Res.* 2016; DOI: 10.1093/nar/gkw408.
